# Neonatal CD8 T-cell Hierarchy Is Distinct from Adults and Is Influenced by Intrinsic T cell Properties in Respiratory Syncytial Virus Infected Mice

**DOI:** 10.1371/journal.ppat.1002377

**Published:** 2011-12-01

**Authors:** Tracy J. Ruckwardt, Allison M. W. Malloy, Emma Gostick, David A. Price, Pradyot Dash, Jennifer L. McClaren, Paul G. Thomas, Barney S. Graham

**Affiliations:** 1 Vaccine Research Center, National Institute of Allergy and Infectious Disease, National Institutes of Health, Bethesda, Maryland, United States of America; 2 Department of Infection, Immunity and Biochemistry, Cardiff University School of Medicine, Cardiff, Wales, United Kingdom; 3 St Jude Children's Research Hospital, Memphis, Tennessee, Unites States of America; University of Massachusetts Medical Center, United States of America

## Abstract

Following respiratory syncytial virus infection of adult CB6F1 hybrid mice, a predictable CD8+ T cell epitope hierarchy is established with a strongly dominant response to a K^d^-restricted peptide (SYIGSINNI) from the M2 protein. The response to K^d^M2_82-90_ is ∼5-fold higher than the response to a subdominant epitope from the M protein (NAITNAKII, D^b^M_187-195_). After infection of neonatal mice, a distinctly different epitope hierarchy emerges with codominant responses to K^d^M2_82-90_ and D^b^M_187-195_. Adoptive transfer of naïve CD8+ T cells from adults into congenic neonates prior to infection indicates that intrinsic CD8+ T cell factors contribute to age-related differences in hierarchy. Epitope-specific precursor frequency differs between adults and neonates and influences, but does not predict the hierarchy following infection. Additionally, dominance of K^d^M2_82-90_ –specific cells does not correlate with TdT activity. Epitope-specific Vβ repertoire usage is more restricted and functional avidity is lower in neonatal mice. The neonatal pattern of codominance changes after infection at 10 days of age, and rapidly shifts to the adult pattern of extreme K^d^M2_82- 90_ -dominance. Thus, the functional properties of T cells are selectively modified by developmental factors in an epitope-specific and age-dependent manner.

## Author Summary

RSV causes yearly winter epidemics of respiratory disease with peak hospitalization rates at 2.5 months of age. Clearance of virus-infected cells depends on CD8 T-cells, and defining mechanisms of CD8 T-cell regulation is essential for understanding RSV disease pathogenesis and guiding therapeutic interventions. CD8 T-cells recognize a virus-infected cell by detecting peptides cleaved from viral proteins that are presented in host cell MHC molecules. The strength of CD8 T-cell response to processed peptide epitopes from the virus commonly assumes a predictable response hierarchy. In adult hybrid mice that have both H-2^b^ and H-2^d^ MHC alleles, the majority of the CD8 T-cell response is targeted at a peptide from the M2 protein and presented by the K^d^ MHC molecule, K^d^M2_82-90_, and a smaller subset of CD8 T-cells respond to a peptide from the M protein, D^b^M_187-195_. Interestingly, when infecting neonatal hybrid mice the dominance pattern is not seen and the D^b^M_187-195_ response is equal to or greater than the K^d^M2_82-90_ response. We show that the adult dominance pattern emerges at 10 days of age and that T cells are modified by developmental factors in an epitope-specific and age-dependent manner. These observations may influence future vaccine design.

## Introduction

Infants are uniquely affected by respiratory syncytial virus (RSV), the leading viral pathogen of the lower respiratory tract in this age group worldwide [1]. In adults RSV is primarily an under-recognized cause of upper respiratory tract illness [2]. RSV causes yearly winter epidemics with most children becoming infected during their first RSV season. Ninety percent of infants are infected by 2 years of age, with the incidence of severe disease peaking between 6 weeks and 6 months [3]. RSV also causes significant morbidity in children and is the number one cause of hospitalization in those under the age of 12 months [4].

Young infants experience increased vulnerability to infectious agents, particularly viral pathogens, suggesting that their T cell-mediated immune responses are different from those in adults [5], [6]. Viruses such as RSV and influenza that cause acute infections in immunocompetent adults often result in protracted illnesses in neonates [2], [7]. These infections ultimately resolve, suggesting delayed viral clearance rather than a persistent inability to clear the offending pathogen. CD8+ T cells play an important role in immunity against viruses through cytokine production and the killing of infected target cells [8], [9] Activation of naïve T lymphocytes and the expression of effector activity by activated CD8+ T cells requires engagement of the T cell receptor (TCR) by viral epitopes presented on MHC class I complexes displayed by antigen-presenting cells (APCs) [10]. Although viral pathogens encode thousands of potentially immunogenic determinants, CD8+ T cell responses are usually targeted against a few viral epitopes. These targeted epitopes generally conform to a hierarchy, with one or two dominant epitopes and several subdominant epitopes [11]. Depending on the nature of the stimulus, however, engagement of the TCR and accessory signaling can result in a variety of outcomes for responding T cells that range from full activation and differentiation through to aborted activation and anergy.

The differences in T cell-mediated immunity between neonates and adults are not well understood. The limited number of human studies performed to date suggest that the neonatal T-cell compartment may be immature [12] and that neonatal CD8+ T cells may require additional stimuli [13]. Murine models have provided further insights into the differences between neonatal and adult CD8+ T cell populations. Studies have shown that neonatal mice have 1-2 log_10_ fewer T cells than adult mice [14], [15]. Furthermore, neonatal TCR chains have a lower frequency of N-nucleotide additions due to a deficiency of terminal deoxynucleotidyl transferase (TdT) until approximately 4 days after birth [16]. Thus, neonatal TCRβ CDR3 loops are shorter by an average of one amino acid [17], [18]. However neonatal mice develop a diverse T cell repertoire in the thymus and periphery that approximates the same order of magnitude compared to adults [14]. In addition, despite differences that would suggest a deficient cytolytic response, a more robust “adult-like”CD8+ T cell response can be generated in certain circumstances, including DNA vaccination and other vaccinations that involve Toll-like receptor (TLR) ligands [19], [20], [21].

Here, we elucidate key differences between adult and neonatal CD8+ T cell responses using a murine model of RSV infection. Responses in adult hybrid CB6F1 mice [22] were compared to responses in neonatal CB6F1 mice with respect to the hierarchy, function, phenotype and clonotypic composition of epitope-specific CD8+ T cell populations. Of the two primary CD8+ T cell populations that typically respond to RSV infection, we find that the K^d^M2_82-90_-dominant CD8+ T cell epitope hierarchy found in adult CB6F1 mice is absent in neonates; instead, the K^d^M2_82-90_ and D^b^M_187-195_ epitope-specific CD8+ T cell responses are numerically codominant. These patterns are maintained throughout primary infection, and sustained during the memory phase. Neonatal CD8+ T cells appear highly functional following stimulation with saturating concentrations of peptide, but have lower functional avidities compared to the corresponding adult cells. The neonatal and adult epitope-specific TCRβ repertoires also differ, but the observed differences in epitope hierarchy are not due to limited TdT activity. We performed the first reported naïve precursor frequency analysis in neonates and found a lower frequency of K^d^M2_82-90_-specific cells, which does not predict the epitope hierarchy established following infection. Rather, a distinct transition time point governs the switch to an adult-like response pattern. Infection prior to day of life 10 results in a codominant response, but infection at or after day of life 10 results in a K^d^M2_82-90_-skewed, adult-like response. Critically, after transfer of naïve adult CD8+ T cells into congenic neonates, the transferred cells respond with the expected adult-like hierarchy while the neonatal host cells display the typical codominant neonatal pattern. These data indicate that factors intrinsic to CD8+ T cell maturation and development can determine the qualitative nature of the effector response and represent the first example of a differential age-dependent epitope-specific CD8+ T cell response hierarchy to the same viral pathogen. Collectively, these findings may have important implications for understanding the pathogenesis of infectious diseases and immunity in neonates.

## Results

### Neonatal CB6F1 mice are less permissive for RSV replication than adult mice

**Figure 1:**
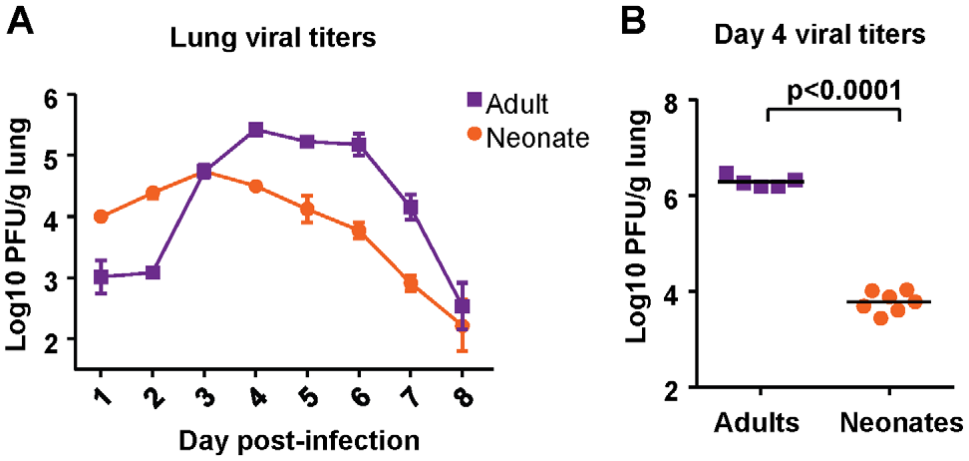
Viral titer kinetics in RSV-infected adult and neonatal mice. (A) Viral titers in the lungs of mice at days 1-8 following infection with 2×10^6^ PFU of RSV. Error bars represent the SEM. (B) Viral titers in the lungs of adult and neonatal mice at day 4after infection with 1×10^7^ PFU of RSV. Horizontal bars represent the mean values. Data are representative of experiments performed twice with 4-8 mice/group.

Viral titer kinetics were performed after infection of both neonatal (7 days old) and adult (6-8 weeks old) CB6F1 mice with 2×10^6^ plaque forming units (PFU) of RSV A2 under isoflurane anesthesia. Titers in the lung were measured at days 1-8 post-infection. Adult mice experienced an eclipse phase early in infection, followed by strong replication in the lung between days 2 and 4 post-infection. Viral load peaked above 5 logs, and dropped rapidly after day 6 post-infection (Figure 1A). Neonatal mice infected with the same dose of virus did not clear the virus as effectively in the early phase, and had higher titers at days 1 and 2 post-infection compared to adults. Replication in neonatal mice was more modest, and peaked at about one log lower compared to replication in adults, after which a gradual tapering of viral titers was observed (Figure 1A).

To see if viral titers depended on the challenge dose, both adult and neonatal mice were infected with 1×10^7^ PFU, and viral titers were measured at day 4 (peak viral titer). Adults infected with a higher dose had a one log increase in viral titer compared to those infected with a lower dose (Figure 1B). However, neonatal mice infected with a high-titer dose did not achieve a viral titer above 4 logs (Figure 1B). Thus, both low dose and high dose infections demonstrated that neonatal CB6F1 mice are less permissive for replication than adults of the same strain.

### Neonatal CD8+ T cells display a different immunodominance profile compared to adult CD8+ T cells

**Figure 2:**
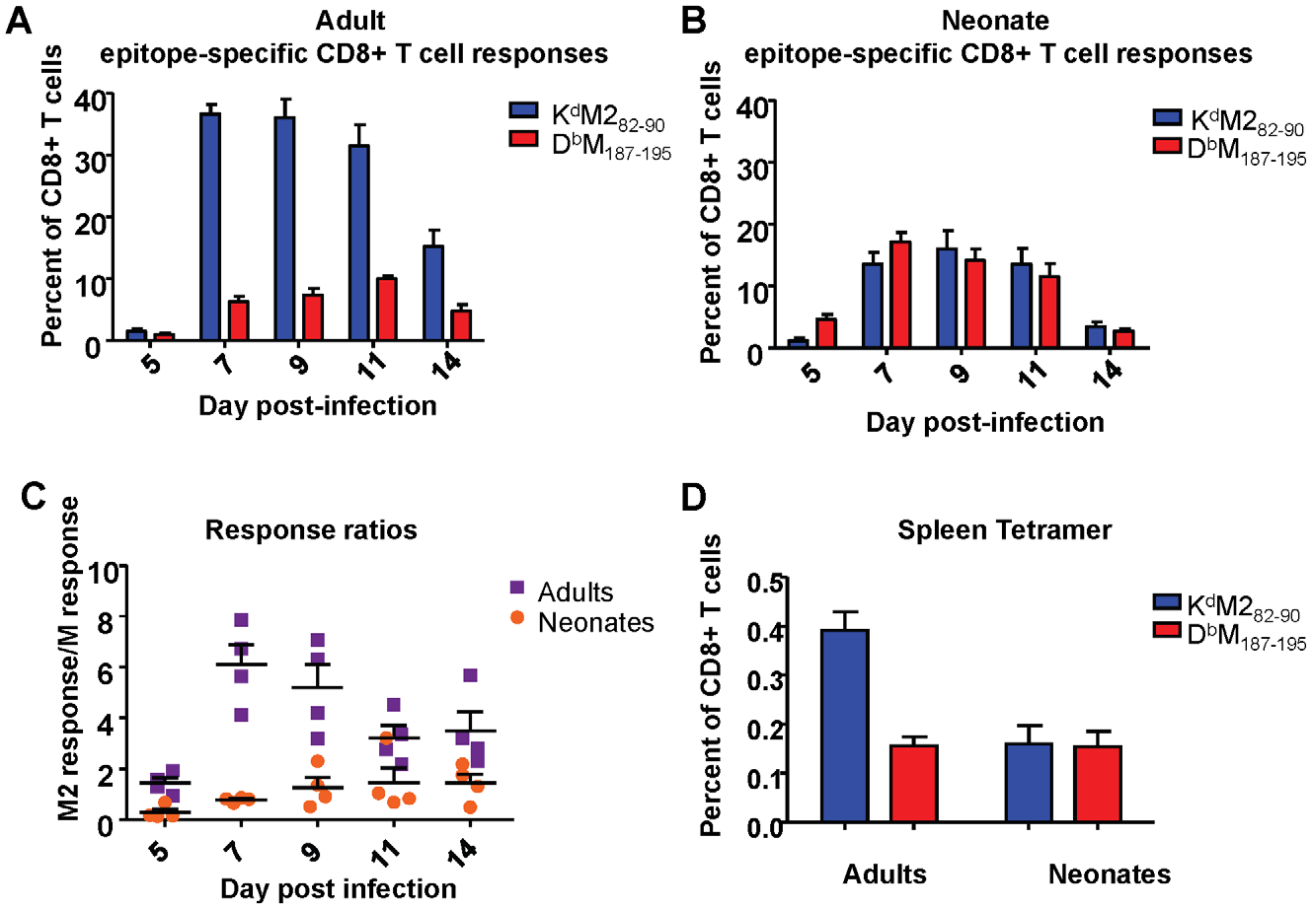
RSV epitope-specific CD8+ T cell responses during acute infection and memory. (A) Tetramer-positive cells specific for K^d^M2_82-90_ and D^b^M_187-195_ were measured in the lung at days 5-14 following infection of adult mice. Percentages of CD3+CD8+ cells staining with the indicated tetramers are shown. (B) Epitope-specific CD8+ T cell responses to K^d^M2_82-90_ and D^b^M_187-195_ measured in the lung at 5-14 days post-infection in neonatal CB6F1 mice. (C) Epitope response ratios (K^d^M2_82-90_/D^b^M_187-195_) measured from lung tissue at days 5-14 post-infection of adult and neonatal mice (D) Epitope-specific memory CD8+ T cell responses in the spleens of mice infected 72 days earlier as adults or neonates. Data are representative of 2-3 independent experiments with 4-8 mice/group. Error bars represent the SEM.

Next, we analyzed CD8+ T cell responses specific for two major viral epitopes in both neonatal and adult CB6F1 mice infected with 2×10^6^ PFU of RSV. We have previously shown that the K^d^M2_82-90_ epitope is immunodominant when compared to the D^b^M_187-195_ epitope in infected adult CB6F1 mice [22]. Adult RSV infected mice showed a strongly dominant K^d^M2_82-90_ response in the lung throughout the course of primary infection (between days 5 and 14, Figure 2A). Mice infected at 7 days of age exhibited lower overall CD8+ T cell responses compared to adults. Surprisingly, in contrast to adult mice, neonates showed a similar (codominant) response to both the K^d^M2_82-90_ and the D^b^M_187-195_ epitopes at all time points following infection (Figure 2B). To further evaluate epitope dominance, the K^d^M2_82-90_ response of each individual mouse was divided by the D^b^M_187-195_ response to yield a response ratio of the two epitopes. The K^d^M2_82-90_-dominant response in adult mice resulted in ratios of 4-6 after day 7 post infection. Codominant neonatal responses resulted in ratios between 0.5 and 1.5 throughout the course of infection (Figure 2C). Differences in the response to acute infection were maintained during the memory phase. Analysis of epitope-specific memory CD8+ T cells 72 days after infection showed a K^d^M2_82-90_-skewed memory response in mice infected as adults (average response ratio  =  2.72), and a codominant response in mice that were infected neonatally (average response ratio  =  1.08)(Figure 2D).

### Neonatal CD8+ T cells are highly functional, but display lower response avidities than adult CD8+ T cells

**Figure 3:**
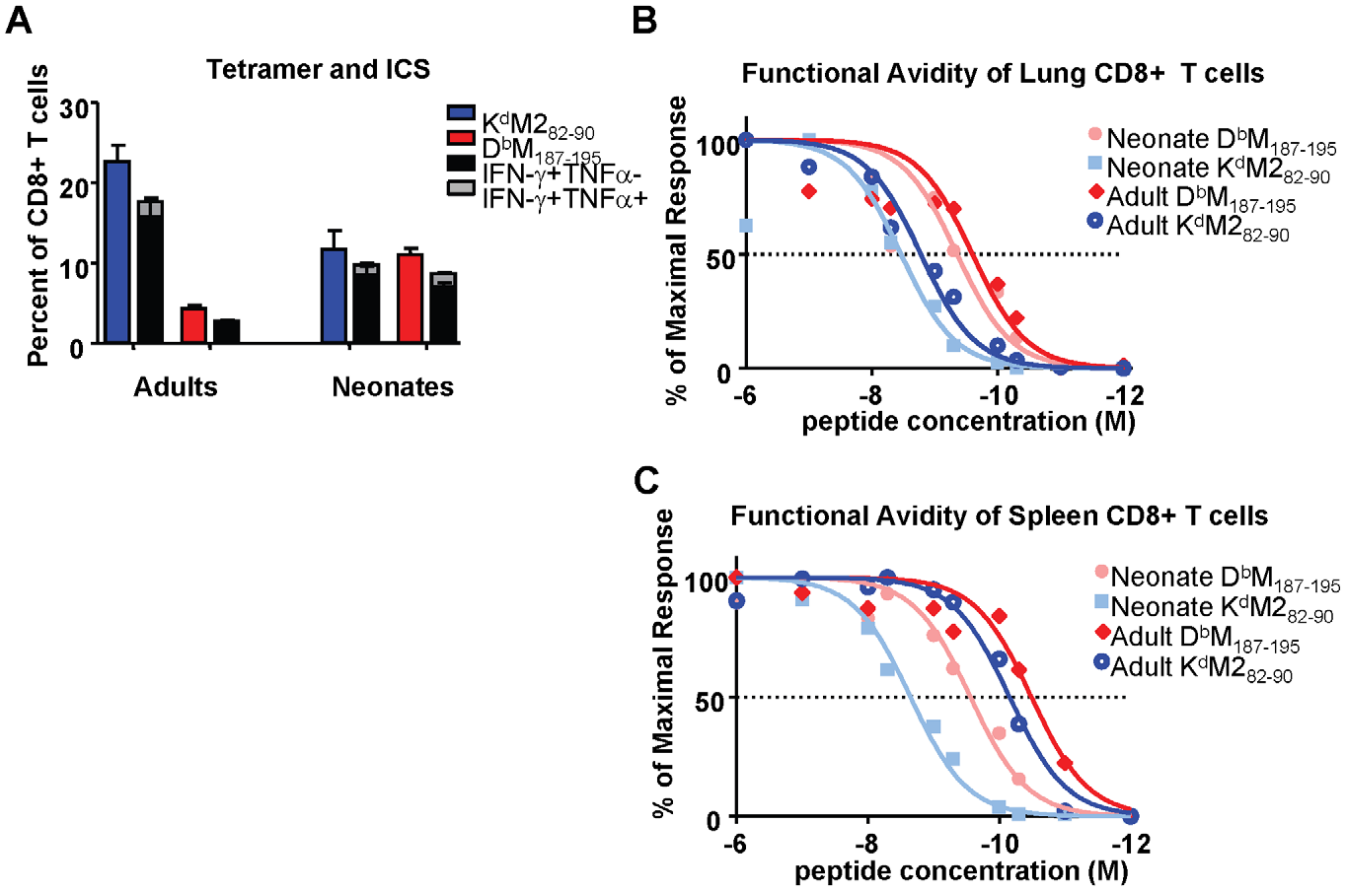
Epitope-specific CD8+ T cell cytokine production and functional avidities in RSV-infected adult and neonatal mice. (A) Frequency and cytokine production by CD8+ T cells specific for K^d^M2_82-90_ and D^b^M_187-195_ in the lungs of RSV-infected adults and neonates. Parallel samples were stained with tetramer and incubated with the M2_82-90_ or M_187-195_ peptide for 5 hours in the presence of the transport inhibitor monensin to measure intracellular cytokine staining by flow cytometry. Tetramer frequencies are indicated by the blue and red bars for the K^d^M2_82-90_ and D^b^M_187-195_ CD8+ T cell populations, respectively. (B) Functional avidities of K^d^M2_82-90_ and D^b^M_187-195_ –specific IFN-γ-producing CD8+ T cells in the lungs of infected adults and neonates. All data were normalized to 100% production at 10^−6^M of each peptide. (C) Functional avidities of K^d^M2_82-90_ and D^b^M_187-195_ specific IFN-γ-producing CD8+ T cells in the spleens of infected adults and neonates. Data represent results from 3-4 independent experiments with 4-7 mice/group. Error bars represent the SEM.

Intracellular cytokine staining (ICS) was performed to evaluate the function of epitope-specific CD8+ T cells from neonatal and adult mice at day 7 after RSV-infection, which represents the peak of the CD8+ T cell response. Tetramer responses were evaluated in parallel with samples that were stimulated with 1×10^−6^M of M2_82-90_, M_187-195_ or flu peptide controls as described in the Materials and Methods. The percentages of tetramer-binding, epitope-specific cells from infected adults and neonates were compared to the percentages of cells that produced cytokine following specific peptide stimulation. Cells were evaluated for the production of IFN-γ, TNF-α and IL-2. However, almost all CD8+ T cells produced either IFN-γ alone, or IFN-γ and TNF-α together upon specific stimulation in both neonates and adults. As we have described previously, the adult response to the K^d^M2_82-90_ epitope was less functional than the response to the D^b^M_187-195_ epitope with regards to cytokine production (i.e. a lower percentage of cells are capable of producing cytokine) [22] (Figure 3A). In contrast, neonatal cells were highly functional upon peptide stimulation, and almost all cells specific for both the K^d^M2_82-90_ and D^b^M_187-195_ produced IFN-γ, or IFN-γ and TNF-α following peptide stimulation (Figure 3A).

The response of CD8+ T cells to stimulation with saturating concentrations of peptide may not reflect either the avidity of the responding populations or the functional response to physiological concentrations of peptide *in vivo*. We therefore assessed the functional avidities of epitope-specific CD8+ T cell populations from both the lungs and spleens of adult and neonatal mice at day 7 post-infection by measuring IFN-γ production following stimulation with peptide concentrations ranging between 1×10^−6^ and 1×10^−12^ M. The percentages of cells producing IFN-γ upon stimulation with each dose of peptide were normalized to the percentages of cells producing cytokine at a saturating peptide concentration (10^−6^M) for each epitope, and log transformation and nonlinear fits were performed using Graphpad PRISM. The functional avidity of each epitope-specific CD8+T cell population was then measured as a half maximal response (concentration of peptide necessary for 50% of the cells to produce IFN-γ). In the lung, adult D^b^M_187-195_-specific cells responded to lower concentrations of cognate peptide (LogEC_50_  =  -9.6) than K^d^M2_82-90_-specific cells (LogEC_50_  =  -8.8), indicating that the subdominant D^b^M_187-195_ response has better functional avidity at the site of infection (Figure 3B). Neonatal responses in the lung demonstrated a similar difference of about one log between the codominant D^b^M_187-195_ and K^d^M2_82-90_ responses (LogEC_50_ of -9.4 and -8.5, respectively), and were overall slightly lower (<0.4 logs) compared to the corresponding functional avidities observed in adults (Figure 3B). A different picture emerged in the spleen, where adult D^b^M_187-195_ and K^d^M2_82-90_-specific CD8+ T cells each responded to considerably lower peptide concentrations than the corresponding cells from the adult lung (LogEC_50_ of -10.5 and -10.2, respectively), while neonatal D^b^M_187-195_ and K^d^M2_82-90_-specific cells were slightly less responsive to peptide (LogEC_50_ of -9.6 and -8.7, respectively) (Figure 3C). The resulting difference between CD8+ T cell responsiveness in adults and neonates was much larger than that observed in the lungs. In both adult and neonatal mice, and in both the lung and the spleen, D^b^M_187-195_-specific CD8+ T cells exhibited higher functional avidities than K^d^M2_82-90_-specific CD8+ T cells.

### Differences in epitope dominance hierarchy between neonates and adults are not related to RSV delivery and replication

**Figure 4:**
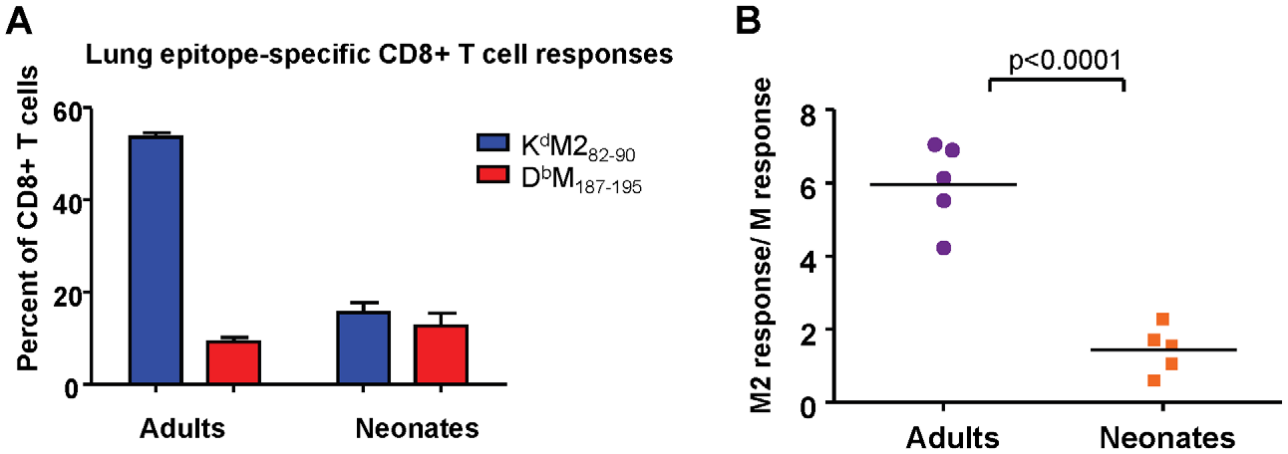
Epitope-specific CD8+ T cell responses following infection with rAd5-MM2. (A) K^d^M2_82-90_ and D^b^M_187-195_ tetramer-binding CD8+ T cells in the lungs of mice infected as adults or neonates 7 days earlier with replication-defective rAd5 vector expressing M and M2 in the form of a fusion protein. (B) Epitope-specific CD8+ T cell response ratios. Data are representative of two independent experiments with 5 mice/group. Error bars represent the SEM.

RSV has several described immunomodulatory strategies, most attributed to the G protein, which may impact CD8+ T cell epitope hierarchy. To evaluate whether the differences in epitope dominance hierarchy between neonatal and adult mice was RSV-specific, the M and M2 antigens from RSV were expressed in the form of a fusion protein from a replication-incompetent rAd5 vector (see Materials and Methods). In this way, both epitopes were equally available and processed from the same protein. Adults and neonates were infected intranasally with 5×10^7^ FFU, and K^d^M2_82-90_ and D^b^M_187-195_ CD8+ T cell responses were measured at day 7 after infection. Consistent with the patterns observed in RSV infection, adult mice displayed a strongly K^d^M2_82-90_-skewed response to rAd-MM2 (Figure 4A), with a response ratio of 6 (Figure 4B). Neonates generated a codominant CD8+ T cell response to rAd-MM2 infection with a response ratio of approximately 1 (Figures 4A and B, p<0.0001). Similar results were observed using a Semliki Forest virus vector to deliver the MM2 fusion protein (data not shown). Thus, the observed differences in epitope hierarchy do not result from immunomanipulation by RSV, but are an intrinsic feature of the age-associated CD8+ T cell response.

### Neonatal and adult epitope-specific CD8+ T cell repertoires are broadly similar

**Figure 5:**
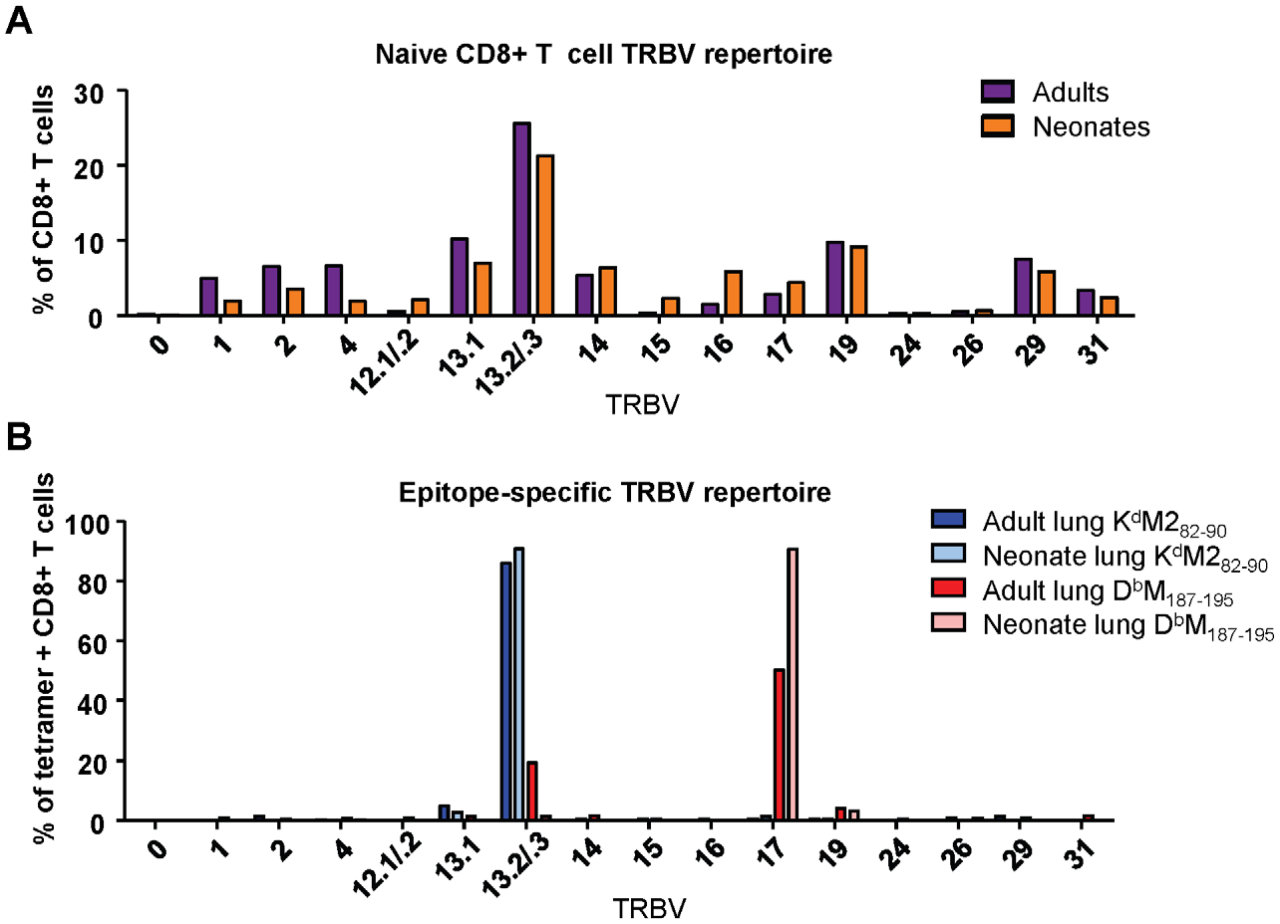
TCR Vβ screening of naïve and epitope-specific CD8+ T cells in adult and neonatal mice. (A) TCR Vβ antibody screen of CD8+ T cells from the spleen of naïve adult and naïve neonatal CB6F1 mice. Samples were stained with surface phenotype antibodies, and a panel of FITC-labeled Vβ-specific antibodies. (B) TCR Vβ antibody screen of epitope-specific (K^d^M2_82-90_ and D^b^M_187-195_) CD8+ T cell populations in the lungs of RSV-infected adults and neonates. Lung lymphocytes were stained with surface phenotype antibodies, epitope-specific tetramer, and Vβ-specific antibodies. All TRBV regions are listed using IMGT nomenclature. Data represents two independent experiments performed with pooled naïve splenic or lung lymphocytes.

Next, we evaluated the naïve repertoire of the CD8+T cell compartment in adult and neonatal mice by staining splenic lymphocytes with antibodies to CD3 and CD8, and a panel of TCR Vβ specific antibodies. Overall, we observed a similar T cell receptor Vβ (TRBV) expression profile in adults and neonates, with some small differences in minor populations (Figure 5A). TRBV13-2/13-3 (IMGT nomenclature,www.imgt.org) (TCR Vβ 8.2/8.1) was the most dominant variable chain expressed in both adult and neonatal naïve CB6F1 mice, followed by TRBV13-1(TCR Vβ 8.3)and TRBV19 (TCR Vβ 6).

We then conducted a similar evaluation of epitope-specific CD8+ T cells by costaining with tetramer and the panel of Vβ-specific antibodies at day 7 after RSV infection. The K^d^M2_82-90_ response was comprised almost exclusively of cells using TRBV13-2/13-3 in both infected adults and neonates. The response to D^b^M_187-195_ was also fairly restricted, with predominant usage of TRBV17 (TCR Vβ 9) in both the adult and neonatal CD8+ T cell populations. However, adult mice showed some usage of TRBV13-2/13-3, which was not observed in the neonatal D^b^M_187-195_-specific response (Figure 5B). Thus, by this crude analysis, TRBV usage within RSV-specific CD8+ T cell populations was largely similar in neonates and adults.

**Figure 6:**
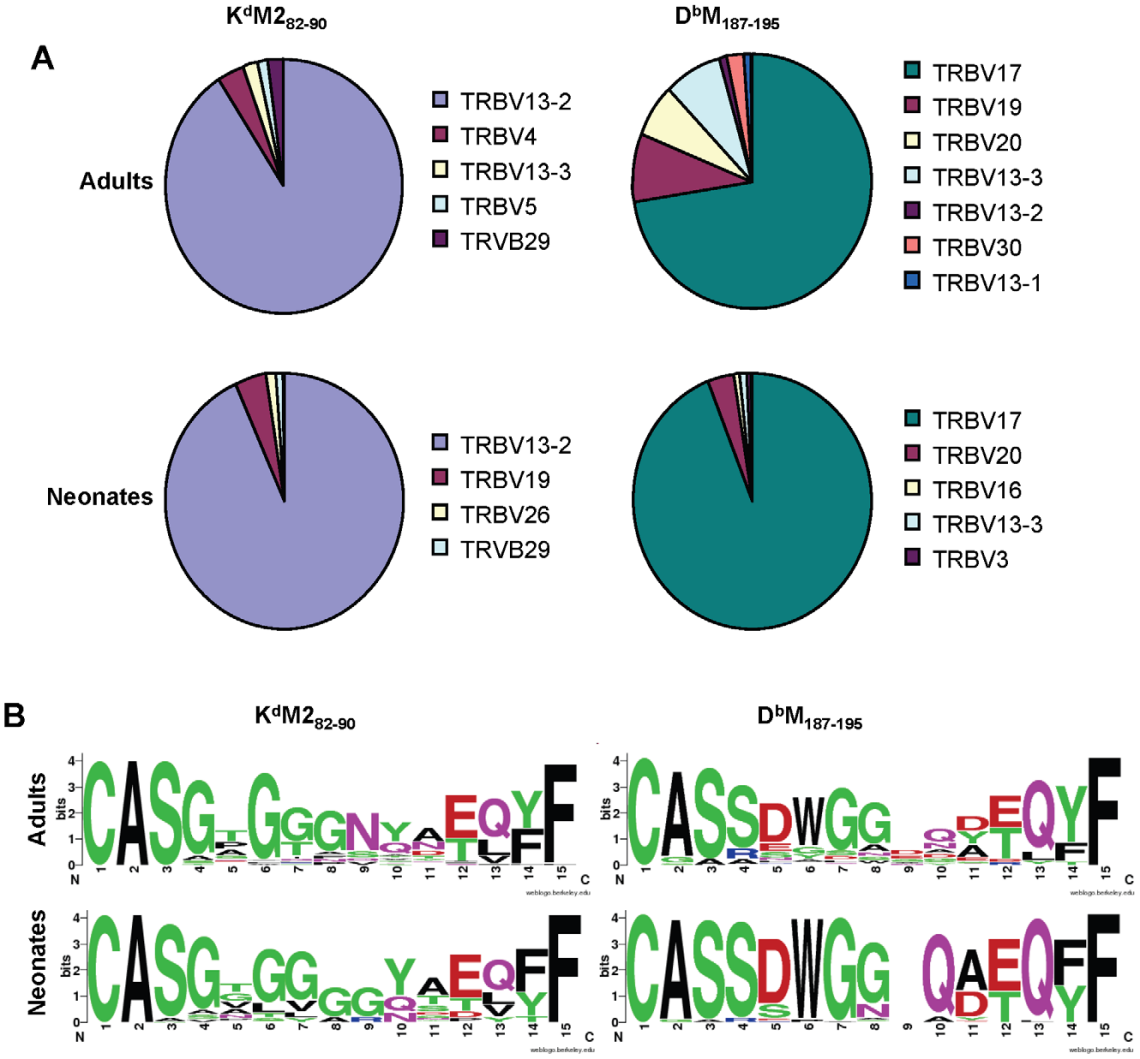
Single cell TCRβ sequences from RSV-specific CD8+ T cell populations. (A) TCR Vβ usage in single CD8+ T cells sorted with K^d^M2_82-90_ (left panels) or D^b^M_187-195_ (right panels) tetramers from RSV-infected adult (upper panels) and neonatal (lower panels) CB6F1 mice (IMGT nomenclature). (B) CDR3β consensus sequences generated on http://weblogo.berkeley.edu/for single CD8+ T cells sorted with K^d^M2_82-90_ (left panels) or D^b^M_187-195_ (right panels) tetramers from RSV-infected adult (upper panels) and neonatal (lower panels) CB6F1 mice. Single tetramer-positive cells were sorted and sequenced from two mice/group/epitope.

To extend our characterization of the RSV-specific CD8+ T cell repertoire, we sequenced TCRs from single tetramer-positive cells sorted by flow cytometry from two infected adult and neonatal mice [23]. Overall, the patterns of TRBV usage observed with antibody staining were mirrored in the single cell sequencing results, with the K^d^M2_82-90_ response consisting primarily of cells using TRBV13-2, and the D^b^M_187-195_-specific response comprising mainly TRBV17-positive cells (Figure 6A). Again, the major difference between adults and neonates pertained to the D^b^M_187-195_ response, which contained a minority of clonotypes with more diverse TRBV usage in adults (Figure 6A).

Single-cell TRBV sequences were also analyzed at the CDR3 level The K^d^M2_82-90_-sorted cells showed considerable diversity across CDR3 amino acid sequences in both adults and neonates, with only a couple of common sequences between the groups (Supplemental Figure S1). The D^b^M_187-195_-sorted cells also showed relatively few common CDR3β sequences between adults and neonates (Supplemental Figure S2); in addition, there was considerably less diversity within the neonatal response, with more than 50% of the sequences obtained having the same CDR3β sequence (CASSDWGGAEQFF). In further analyses, we generated consensus sequences for the CDR3β regions (http://weblogo.berkeley.edu/). Despite considerable diversity, the consensus CDR3β sequences for the responses to the K^d^M2_82-90_ epitope were similar between adults and neonates and featured the highly predominant usage of central glycine residues (Figure 6B). Similarly, CDR3β motifs between adult and neonatal D^b^M_187-195_-specific cells were largely similar. Our past work has shown that the adult D^b^M_187-195_ response contains a relatively conserved DWG motif [24], which was even more prominent in the neonatal consensus sequence due the relatively restricted use of a DWG containing CDR3β (Figure 6B).

### Codominance in the neonatal repertoire is not due to low/limited TdT activity during early life

**Figure 7:**
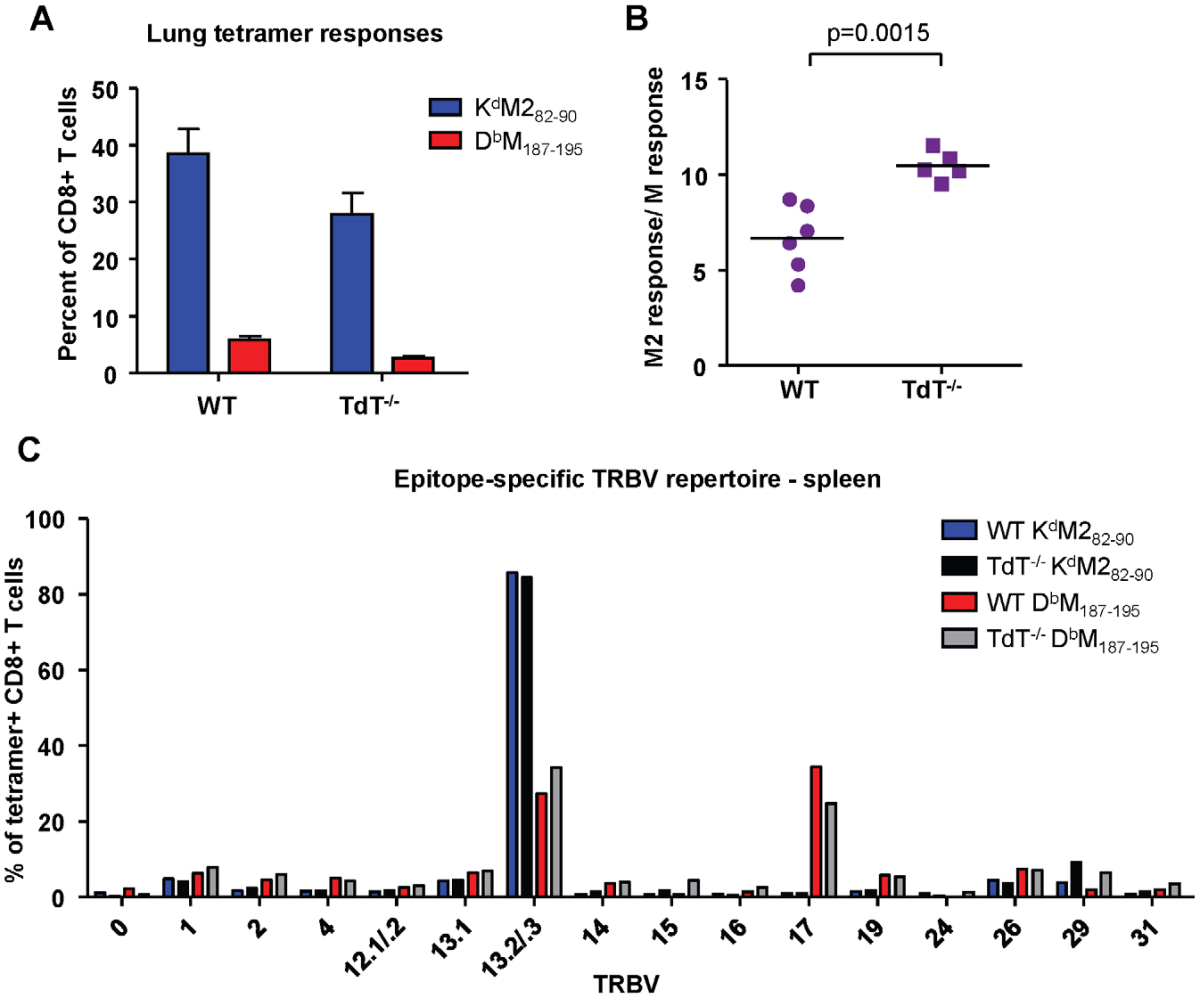
Epitope-specific CD8+ T cell responses in wild-type and TdT^-/-^ adult CB6F1 mice. (A) K^d^M2_82-90_ and D^b^M_187-195_ tetramer-positive CD3+CD8+ in the lungs at day 7 post-infection of wild-type (WT) and TdT deficient (TdT^-/-^) CB6F1 mice. (B) Epitope-specific CD8+ T cell response ratios in infected WT and TdT^-/-^ mice. (C) TCR Vβ antibody screening of pooled tetramer-positive CD8+ T cells from the spleen of infected WT and TdT^-/-^ mice (IMGT nomenclature). Data in panels (A) and (B) are representative of two independent experiments with 5-6 mice/group. Error bars represent the SEM.

Terminal deoxynucleotidyl Transferase (TdT), the enzyme responsible for all non-template nucleotide additions within the CDR3 region, is not expressed until 4-7 days after birth in neonatal mice. Consistent with lower TdT activity, neonatal epitope-specific CD8+ T cell populations had slightly shorter CDR3β amino acid lengths compared to those of adults (K^d^M2_82-90_: 12.1 vs. 13.1, D^b^M_187-195_: 13.2 vs. 13.6 respectively; data not shown). To determine if the epitope dominance disparity between adults and neonates is due to low or limited TdT expression in early life, we infected adult wild-type and TdT knockout (TdT^-/-^) CB6F1 mice. Overall, epitope-specific responses were lower in TdT^-/-^ mice than they were in wild-type mice (Figure 7A). The K^d^M2_82-90_/D^b^M_187-195_ response ratio in TdT^-/-^ mice was significantly higher than in wild-type mice, indicating a more K^d^M2_82-90_-skewed response (Figure 7B, p = 0.0015). Furthermore, CDR3β sequences obtained from TdT^-/-^ mice were on average 1 and 2 amino acids shorter for the D^b^M_187-195_-specific and K^d^M2_82-90_-specific CD8+ T cell responses, respectively (data not shown). These data indicate that a lack of TdT activity is not responsible for the codominant epitope hierarchy observed following RSV infection of neonatal mice. TCR Vβ usage by tetramer-positive CD8+ T cells from infected wild-type and TdT^-/-^ adult mice was similar for both the K^d^M2_82-90_-specific and D^b^M_187-195_-specific responses (Figure 7C).

### Precursor frequencies of epitope-specific CD8+ T cells in naïve adult and neonatal CB6F1 mice does not predict response hierarchy after infection

**Figure 8:**
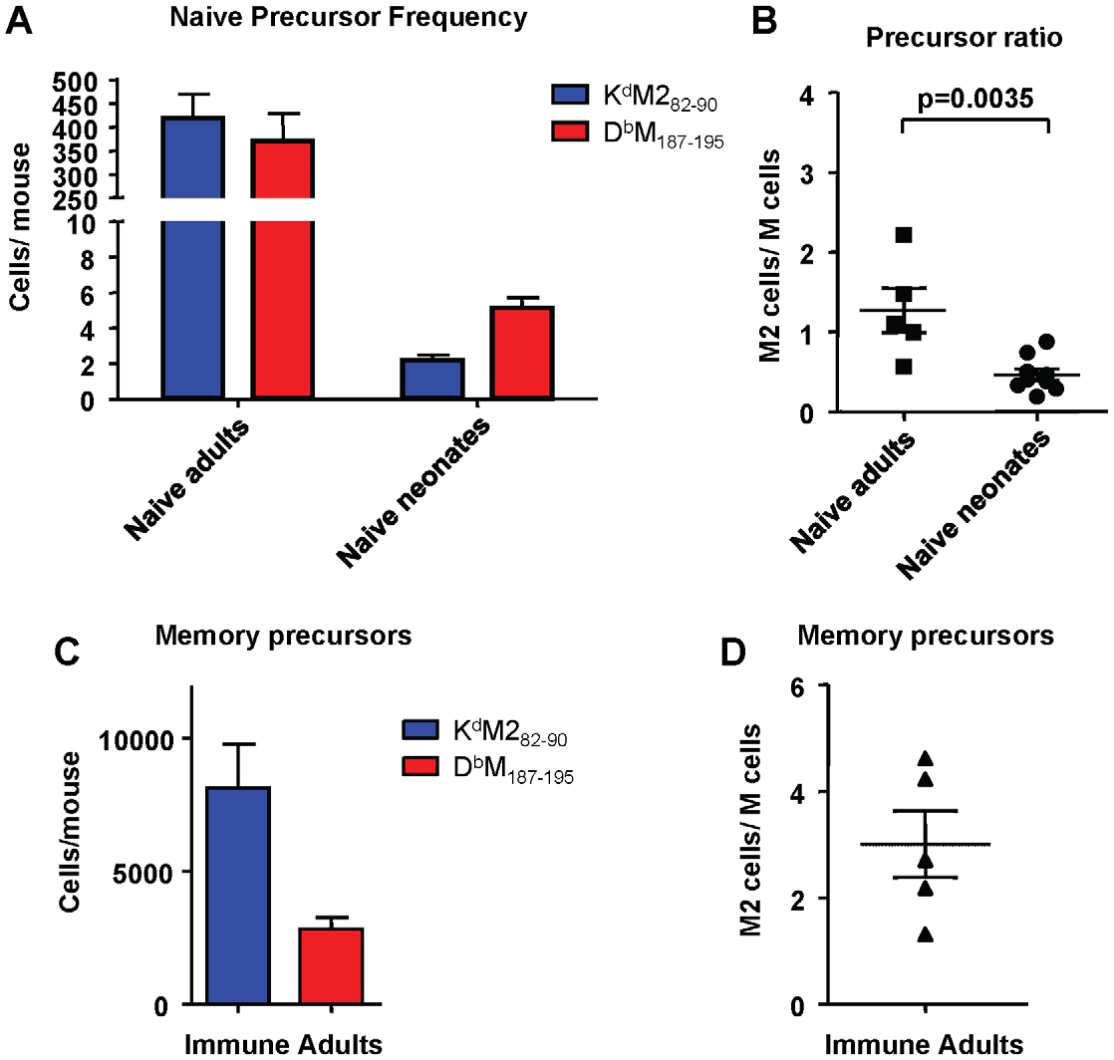
Epitope-specific CD8+ T cell precursor frequency analysis in adult and neonatal mice. (A) Precursor frequencies of epitope-specific CD8+ T cells in naïve adult and neonatal mice as determined by the double tetramer enrichment method. (B) K^d^M2_82-90_/D^b^M_187-195_ precursor frequency ratios in naïve adults and neonates. (C) Epitope-specific memory CD8+ T cell precursor frequencies in adult mice infected one month previously with RSV. (D) Memory CD8+ T cell precursor ratios. Data are representative of several independent experiments with 5-12 mice/group. Error bars represent the SEM.

To determine if the differences in response hierarchy between adult and neonatal mice was due to a difference in epitope-specific CD8+ T cell precursor frequencies, we enumerated naive precursors using a double tetramer enrichment protocol as described in the Materials and Methods. For adult mice, spleens and macroscopic lymph nodes were harvested; for neonatal mice only spleens were harvested, and 8-12 spleens were pooled for each sample. Cells from naïve adult and neonatal CB6F1 mice were processed at the same time as samples from OT-I/RAG1^-/-^ mice (negative controls), and samples from immune/memory CB6F1 mice that were infected as adults at least one month previously. Data were generated with two sets of tetramers for each epitope: one set was purchased from Beckman Coulter, and the second set was produced in-house as described previously [25]. Although the data were consistent within each set of tetramers, some differences were observed between sets. Representative raw data are shown in Supplemental Figures S3 and S4. Data generated using the in-house tetramers appeared more comparable to published data using the double tetramer method, with positive cells staining equally with each tetramer and forming a clean diagonal; for this reason the in-house tetramers were used for the data presented in Figure 8. Despite the K^d^M2_82-90_-skewed response observed following RSV infection, naïve adult CB6F1 mice had similar numbers of precursors specific for the K^d^M2_82-90_ and D^b^M_187-195_ epitopes (average of 419 vs. 370 cells per mouse, respectively; p = 0.54; Figure 8A), with a resulting median ratio of 1.27 (Figure 8B). In contrast, naïve neonatal CB6F1 had significantly fewer precursors specific for K^d^M2_82-90_ compared to D^b^M_187-195_ (average of 2.2 vs. 5.2 cells per neonate, respectively; p = 0.0003; Figure 8A). The median ratio of K^d^M2_82-90_/D^b^M_187-195_-specific CD8+ T cells in naïve neonates was 0.46 (Figure 8B). As expected, OT-I/RAG1^-/-^ had no precursors specific for either K^d^M2_82-90_ or D^b^M_187-195._ Further, as expected based on our staining results for memory CD8+ T cell responses, mice infected at least one month prior to analysis had significantly higher numbers of precursors specific for K^d^M2_82-90_ (average of 8114 cells/mouse) compared to D^b^M_187-195_ (average of 2811 cells/mouse; Figure 8C), with a median response ratio of 3.01 (Figure 8D); these data mirror the tetramer straining results obtained with memory populations in immune mice (Figure 2D). For comparison, data obtained using the commercial tetramers to enrich cells from the same groups of mice are presented in Supplemental Figure S5.

### Age at infection determines CD8+ T cell immunodominance in RSV infected mice

**Figure 9:**
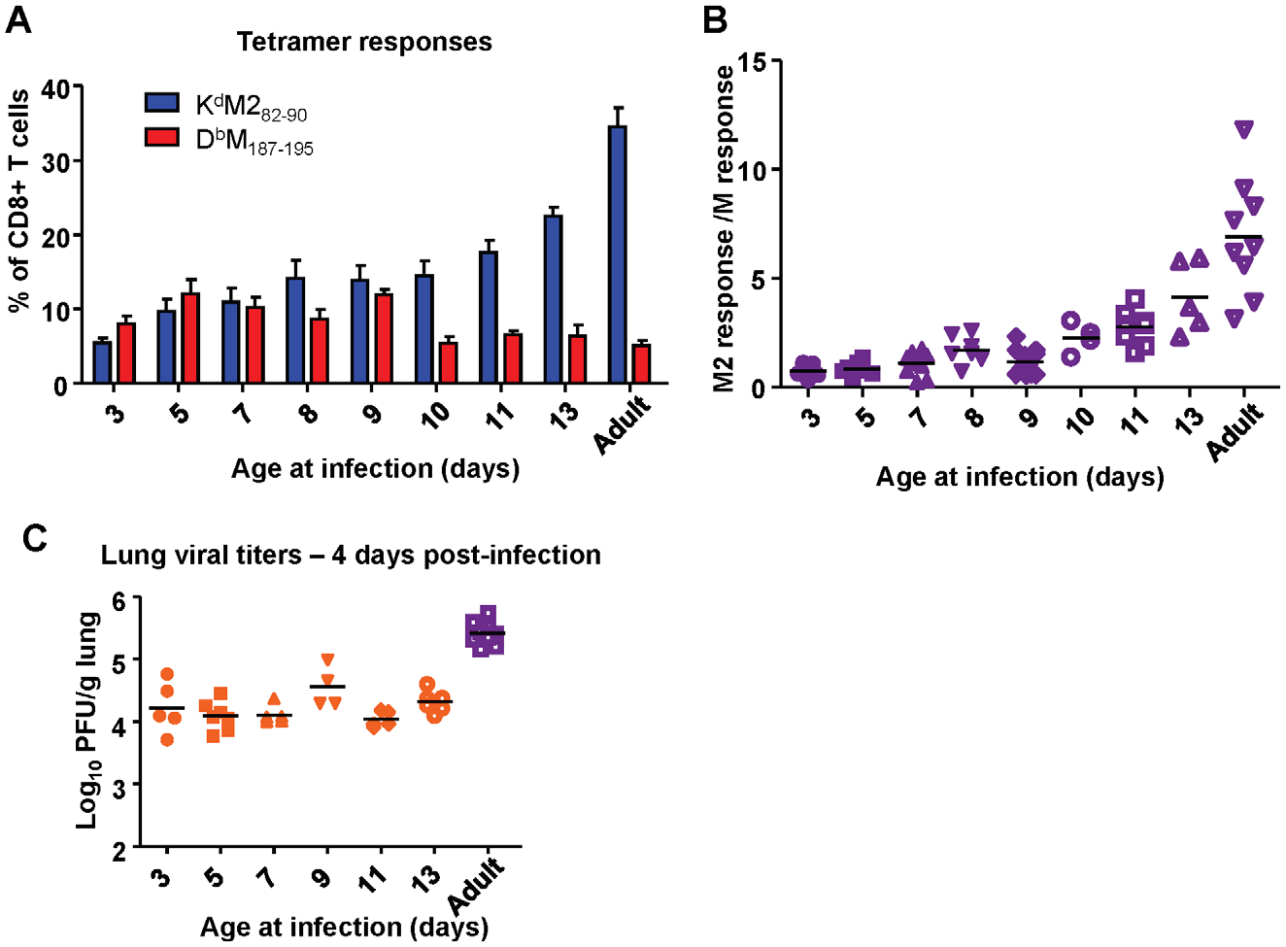
Age at infection determines CD8+ T cell epitope hierarchy. (A) K^d^M2_82-90_ and D^b^M_187-195_ epitope-specific CD8+ T cell responses determined by tetramer staining at day 7 post-infection of mice infected with RSV at the indicated day of life. (B) Epitope-specific CD8+ T cell response ratios at day 7 post-infection of mice infected with RSV at the indicated day of life. (C) Viral titers in the lung at day 4 post-infection of mice infected with RSV at the indicated day of life. Data represent two independent experiments with 4-8 mice/group. Error bars are the SEM.

Next, we asked when the epitope hierarchy shifted by infecting mice between the ages of 3 days of life and 13 days of life and measuring CD8+ T cell responses at day 7 post-infection. Mice infected before day of life 10 exhibited codominant CD8+ T cell responses to K^d^M2_82-90 and_ D^b^M_187-195_. Starting most notably in mice infected at 10 days of life, however, the response became increasingly K^d^M2_82-90_-dominant. This pattern continued through mice infected at day of life 13 into adulthood (Figure 9A) and is clearly visualized as an upward trend on the response ratio graph beginning with mice infected at day of life 10 (Figure 9B). Changes in epitope hierarchy in mice infected at days of life 3 through 13 were not associated with viral titer increases (Figure 9C).

### Intrinsic CD8+ T cell factors contribute to epitope hierarchy post-infection

**Figure 10:**
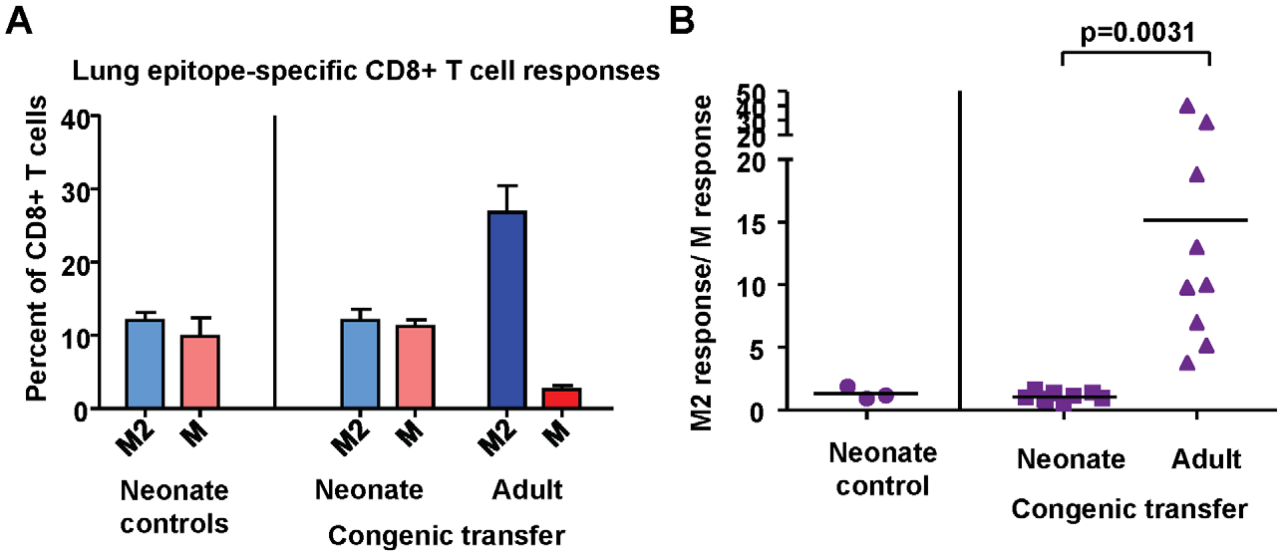
Intrinsic CD8+ T cell factors contribute to epitope hierarchy post-infection. (A) K^d^M2_82-90_ and D^b^M_187-195_-specific CD8+ T cells in the lungs of control neonatal mice, and neonatal mice that received congenic transfer of naïve adult CD8+ T cells. (B) Epitope-specific CD8+ T cell response ratios in control neonatal mice and neonatal mice that received congenic transfer of naïve adult CD8+ T cells. Data are representative of several independent experiments with 3-10 mice/group. Error bars represent the SEM.

To assess the contribution of intrinsic CD8+ T cell factors to the establishment of epitope hierarchy following infection, naïve adult CD8+ T cells were adoptively transferred intra-peritoneal into naïve congenic neonatal mice 2–3 days prior to infection with RSV. Spleen and lymph node CD8+ T cells were purified by untouched isolation from two adults for transfer into each neonate; the final number of transferred CD8+ T cells was between 7×10^6^ and 1.5×10^7^ per neonate. The CD8+ T cell responses of adult (transferred) and neonatal (endogenous) cells were evaluated at day 7 post-infection (Supplemental Figure S6). The endogenous CD8+ T cell response in neonatal mice in the absence of adoptive transfer was codominant for K^d^M2_82-90_ and D^b^M_187-195_, with an average response ratio of 1.3 (Figure 10A and B). In mice that received naïve adult CD8+ T cells, the endogenous response was similarly codominant with an average response ratio of 1.2. However, the adult transferred cells within the same animals generated a strongly K^d^M2_82-90_ skewed response with an average ratio of 15.2; p = 0.0031; Figure 10A and B) similar to that observed following infection of adult mice. These data indicate that intrinsic CD8+ T cell factors play an important role in the establishment of epitope hierarchy following infection.

## Discussion

Respiratory syncytial virus is a significant cause of morbidity and mortality in infancy, with most severe disease occurring before 6 months of age. Currently, prophylactic treatment with the humanized monoclonal antibody Synagis is the only licensed intervention for RSV and is used to prevent hospitalization caused by severe RSV disease. However, passive antibody treatment is expensive, only partially effective, and available only to those at highest risk. There is a great need for a preventive vaccine, or effective therapeutic treatment strategies. To be of most benefit, an RSV vaccine would need to be given early in life, and overcome many obstacles associated with the generation of effective immunity during infancy [26]. Induction of effective immunity to RSV is challenging due to the failure of natural immunity to protect against reinfection. Vaccine safety is also a concern because of the history of vaccine-enhanced disease following natural infection after a formalin-inactivated vaccine given in the early 1960s [27], [28]. These challenges highlight the critical significance of understanding neonatal adaptive immune responses during both infection and vaccination.

Several studies have demonstrated the importance of the age of infection with regard to the immune response to RSV [29], [30], [31], [32], [33], [34], [35], [36]. Studies in neonatal mice have focused on the influence that neonatal infection has on the immune response to subsequent reinfection with RSV. It has been established that when neonatal BALB/c mice are infected prior to 7 days of age, they experience enhanced disease following reinfection as adults. This disease enhancement has been associated with increased airway hyperresponsiveness, increased airway inflammatory cell recruitment and immunopathology, mucus hyperproduction, eosinophil recruitment, and enhanced Th2 cytokine production [30], [31], [32], [33], [34], [35]. Additionally, infecting neonates with recombinant RSV expressing IFN-γ was found to abrogate disease enhancement, while RSV expressing IL-4 resulted in further enhancement of Th2 responses and eosinophilia [37]. Importantly, in all studies, disease enhancement is not observed following reinfection of mice initially infected as adults. These studies in BALB/c mice provide an example of how early infection can modify and shape subsequent responses to reinfection or other airway insults. These studies also recapitulate some facets of human infection, where severe disease early in life has been linked to the development of childhood wheezing [38] and vaccination during infancy caused enhanced disease following natural infection [28].

As in humans, the CD8+ T cell response in RSV-infected mice is known to play a critical role in viral clearance. Using CB6F1 hybrid mice, the expression of both d- and b- MHC haplotypes creates a more complex phenotype and increases the number of T cell responses that can be measured. In BALB/c mice, the K^d^M2_82-90_ response is so dominant that other responses contribute very little to the outcome of infection. In CB6F1 mice, a reproducible epitope hierarchy is established following RSV infection. The K^d^M2_82-90_ response initially described in the BALB/c parent strain dominates, with an approximately 5-fold lower response to the D^b^M_187-195_ epitope initially described as dominant in the C57BL/6 parent strain [22], [39]. In striking contrast, mice infected as neonates were found to have codominant responses to K^d^M2_82-90_ and D^b^M_187-195_ throughout the course of primary infection, which were maintained in the memory phase. It is generally accepted that neonatal CD8+ T cell responses are lower than adult responses, and this has been described for the K^d^M2_82-90_ epitope following neonatal infection of BALB/c [34]. While we also measure lower K^d^M2_82-90_ responses in CB6F1 neonates, the response to the D^b^M_187-195_ epitope is higher in infected neonates than adults. This observation demonstrates that the ability of neonates to generate CD8+ T cell responses is epitope-dependent and in some cases, may be superior to adult T cell responses. We found that the epitope hierarchy makes a radical shift when mice are infected between 3 and 13 days of age, with the emergence of K^d^M2_82-90_ dominance starting after infection at day of life 9. The dominance pattern skews dramatically for mice infected between days 9 and 13 and is associated with a lowering of the response to the D^b^M_187-195_ epitope and a significant increase in the response to K^d^M2_82-90_. It is likely that dampening of the D^b^M_187-195_ response following emergence of the dominant K^d^M2_82-90_ response reflects immunodomination by this epitope, which we have described in detail in adult CB6F1mice [40]. The relatively low K^d^M2_82-90_ response generated in younger neonates may not reach a magnitude or functional activity sufficient for immunodomination of D^b^M_187-195_.

Functionally, neonatal CD8+ T cells are adept at producing effector cytokines following stimulation with saturating concentrations of cognate peptide. They were as proficient, if not more, in the production of IFN-γ and TNF-α as compared to CD8+ T cell responses generated in the adult. This suggestion of “adult-like” function may be deceptive, however, as peptide titrations showed clearly lower functional avidities for CD8+ T cell responses generated in the neonate, which may play a role in their responsiveness *in vivo*. This difference was particularly apparent in the spleen, where approximately one log more peptide was required to reach a half maximal response in neonatal CD8+ T cells. In both adults and neonates, D^b^M_187-195_-specific cells were found to have higher functional avidities than K^d^M2_82-90_-specific cells, a property that appears to be independent of epitope hierarchy.

Factors known to influence CD8+ T cell epitope dominance fall into three main categories. The first is antigen processing and presentation and includes factors involved in peptide liberation, transport, and class I binding affinity. The second involves characteristics inherent to the CD8+ T cell response such as the T cell repertoire and precursor frequency, and the ability of cells to respond to stimulation by activation and proliferation. Finally, regulation of CD8+ T cell responses, either by other CD8+ T cells (immunodomination) or by regulatory T cells can play a role in the establishment of epitope hierarchy. Adoptive transfer experiments in which adult naïve CD8+ T cells were transferred into neonates gave us the ability to study the response of both neonatal and adult cells within the same infected host. The results of these experiments heavily suggested that intrinsic CD8+ T cell factors help dictate the dominance patterns we observed following RSV infection. We investigated several CD8+ T cell factors that may be involved, starting with characterization of the TCR Vβ repertoire in naïve and infected mice. Despite the dramatic difference in epitope response hierarchy, neonatal responses were surprisingly similar to responses seen in adults in many ways, particularly when responses were studied at the level of TCR Vβ protein expression. Further analysis by single-cell clonotyping was necessary to identify differences at the CDR3β amino acid level, and showed overall less diversity in the neonatal response, particularly for the D^b^M_187-195_ epitope. Despite some sequence and diversity differences, however, the general motifs within K^d^M2_82-90_ and D^b^M_187-195_ CDR3βs were similar between adults and neonates. The impact that these relatively subtle repertoire differences between adults and neonates exert on epitope hierarchy is a subject of further investigation.

A lack of TdT activity in early life has been found to be responsible for shaping the murine neonatal repertoire [41], [42], and neonatal CD8+ T cell responses to infection can consist of shorter CDR3 sequences than those of adults [43]. We hypothesized that TdT may play a role in epitope hierarchy differences between adults and neonates, and infected adult TdT^-/-^ CB6F1 mice to address this possibility. RSV-infected TdT^-/-^ animals had an overall lower CD8+ T cell response, but the relative dominance of the K^d^M2_82-90_-specific response was greater than in wild-type mice, indicating that TdT deficiency does not favor the D^b^M_187-195_ response or account for codominance in infected neonates. Additionally, the TCR Vβ repertoire within both the K^d^M2_82-90_ and the D^b^M_187-195_ response was similar between wild-type and TdT^-/-^ animals.

Precursor frequency is another CD8+ T cell factor that has been found to correlate with epitope hierarchy post-infection [44], [45], [46]. Here, we describe the first reported enumeration of precursor frequencies in neonatal mice. Unlike in adults, lymph nodes cannot be acquired from naïve neonatal mice. The availability of only spleen tissue and the relative lymphopenia of neonates necessitated pooling of at least 8 neonates to work with sufficient cell numbers. Technical constraints in both adults and neonates imply that naïve precursor frequencies are an underestimate in each case, but relative comparisons between epitopes offers reproducible and meaningful results. We analyzed each cell population independently using the same tetramer conjugated to two different fluorochromes simultaneously. Consistent and expected results were seen for each set with regard to the negative controls, and the ratio of K^d^M2_82-90_/D^b^M_187-195_ cells seen in immune mice. While neonates had a more D^b^M_187-195_-skewed population of precursors than adults, precursor frequency did not predict the final epitope hierarchy post-infection. This interpretation was consistent with data generated with either in-house or commercial tetramers. Adult CB6F1 have as many, if not more D^b^M_187-195_-specific precursors, yet generate a severely K^d^M2_82-90_-skewed response. Similarly, the presence of more precursors specific for D^b^M_187-195_ than for K^d^M2_82-90_ in the neonate does not lead to dominance of the D^b^M_187-195_ response. La Gruta *et al.*, have reported that precursor frequency is unrelated to the immunodominance hierarchy observed in adult mice following infection with influenza A virus, and suggest that subdominance is a consequence of inefficient cell recruitment and clonal expansion [47]. It is likely that immunodominance in the RSV model is influenced by a combination of precursor frequencies and differing abilities of naïve CD8+ T cells to be recruited and proliferate. Figure2A clearly illustrates a large net proliferative advantage for K^d^M2_82-90_-specific cells over D^b^M_187-195_-specific cells between days 5 and 7 post-infection in adult mice. The basis for this difference in net frequency of tetramer-positive adult T cells in the lung is currently being explored along with other factors including antigen processing and presentation, CD8+ T cell regulation, and the influence of MHC I-peptide epitope structure and TCR affinity on the functional T cell hierarchy.

In summary, we have described differences in epitope dominance between adult and neonatal CD8+ T cell responses, with associated differences in TCR diversity, functional avidity, and precursor frequency. We show that there are intrinsic properties of adult T cells that result in distinct functional responses in an epitope-dependent manner. The factors that account for the dramatic shift in T cell function between days 9 and 10 of life are the subject of ongoing investigation. This phenomenon is unlikely to be RSV-specific, and we believe that epitope dominance disparities due to age are likely to exist in other mouse models and in humans. A better understanding of the differences between how adult and neonatal responses are generated is of critical importance. The well-known plasticity of the neonatal response can be powerfully manipulated, but immune response patterns in neonates cannot necessarily be predicted by responses in adults. Therefore care must be taken to ensure that optimal effectiveness and safety of vaccine-induced immunity is achieved in neonates.

## Materials and Methods

### Ethics statement

All mice used in this study and analysis were maintained according to the guidelines of the NIH Guide to the Care and Use of Laboratory Animals and the approval of the Animal Care and Use Committee of the Vaccine Research Center (VRC), National Institute of Allergy and Infectious Diseases at the National Institute of Health. All mice were housed in a facility fully accredited by the Association for Assessment and Accreditation of Laboratory Animal Care International (AAALAC). All procedures were conducted in strict accordance with all relevant federal and National Institutes of Health guidelines and regulations.

### Mice and RSV infections

BALB/c (female) and C57BL/6 (male) breeders were purchased from Jackson Labs and pups were obtained by time-mating. Adult (8–10 weeks old) female CB6F1/J mice (Jackson Labs, Bar Harbor, ME or bred in-house) were used. TdT^-/-^ mice on BALB/c and C57BL/6 backgrounds were generously provided by Jonathan Yewdell (NIAID, NIH) and Ann Feeney (Scripps), respectively. CB6F1 TdT^-/-^ mice were bred in-house. Thy1.1+ C57BL/6 mice were obtained from Jackson (B6.PL-Thy1a/CyJ), Thy1.1+ BALB/c mice were a generous gift from Jonathan Yewdell, and Thy1.1+ CB6F1 mice were bred in house. OT-I/RAG-1^-/-^ mice were purchased from Taconic and bred in-house. All mice were housed in our animal care facility at NIAID under specific, pathogen-free conditions, and maintained on standard rodent chow and water supplied *ad libitum*. All studies were reviewed and approved by the NIH Animal Care and Use Committee. Mice were anesthetized using isoflurane (3%) prior to intranasal inoculation with 2×10^6^ PFU live RSV in 10% EMEM (100 µl for adults, 25–35 µl for neonates). Neonatal mice were infected at day 7 of life unless stated otherwise. All mice were euthanized by lethal injection with pentobarbital (250 mg/kg).

### Plaque assays

Mice were sacrificed and lung tissue was removed and quick-frozen in 10% EMEM. Thawed tissues were kept chilled while individual samples were ground using a GentleMACS machine (Miltenyi, Germany) on program Lung 02. Samples were centrifuged, and dilutions of clarified supernatant were inoculated on 80% confluent HEp-2 cell monolayers in triplicate and overlaid with 0.75% methyl cellulose in 10% EMEM. After incubation for 4 days at 37°C, the monolayers were fixed with 10% buffered formalin and stained with hematoxylin and eosin. Plaques were counted and expressed as log_10_ PFU/gram of tissue. The limit of detection was 1.8 log_10_ PFU/gram of tissue.

### Adenovirus MM2

A replication-defective adenovirus expressing a fusion protein of M and M2 was constructed using sequences for RSV-A2 (M, NCBI accession number AAB86660; M2, NCBI sequence number AAB86677), which were codon optimized for expression in humans and mice using the GeneOptimizer technology from GeneArt. The rAd5-MM2 vector was produced by GenVec (Gaithersburg, MD) using complementing mammalian cells (293-ORF6) as described previously [48], and 5×10^7^ focus forming units (FFU) were given intranasally to adults and neonates under isoflurane anesthesia.

### Synthetic peptides

RSV M2_82-90_ (SYIGSINNI) and RSV M_187-195_ (NAITNAKII) peptides were derived from the RSV M2 and M proteins, respectively. The H2-K^d^-binding influenza virus A/Puerto Rico/8/34 nucleoprotein NP_147-155_ (TYQRTRALV) peptide, and H2-D^b^-binding influenza A/Puerto Rico/8/34 NP_366-374_ (ASNENMETM) peptide were used as negative controls. All peptides were synthesized by Anaspec, Inc. (San Jose, CA), and confirmed to be >95% pure by analytical high-performance liquid chromatography at the NIAID peptide core facility (Bethesda, MD).

### Tetramer, TCR Vβ staining, intracellular cytokine staining (ICS) and functional avidity assays

Mice were sacrificed and lung and/or spleen tissues were harvested at the indicated times post-infection. Lung and spleen tissues were disrupted by tissue dissociation using a GentleMACS machine (Miltenyi). Lymphocytes were purified using Fico- LITE at room temperature, washed, then resuspended in 10% RPMI. For ICS, lymphocytes were incubated at 37°C for 5 hours with 1 µM of the appropriate peptide, 1 µg/ml of co-stimulatory antibodies against CD28 and CD49d, and 1 µg/ml of monensin. After incubation, cells were surface stained with fluorochrome-conjugated antibodies against CD3 (145-2C11), CD4 (GK1.5), and CD8 (2.43) then fixed and permeabilized using an intracellular cytokine staining kit according to the manufacturer's instructions (BD, San Diego, CA). Intracellular stains were performed with labeled antibodies to IFN-γ (XMG1.2), IL-2 (JES6-5H4) and TNF-α(MP6-XT22) for 20 min at 4°C. For tetramer analysis, cells were surface stained with K^d^M2_82-90_ or D^b^M_187-195_ tetramer (Beckman Coulter, San Diego, CA) together with labeled antibodies specific for CD3, CD4, and CD8. Samples for TCR Vβ usage analysis also included an antibody from the TCR Vβ screening panel (BD). After staining, cells were washed and analyzed by flow cytometry. Samples were collected on an LSR-II flow cytometer (BD, San Jose, CA) and data were analyzed using FlowJo version 8.8.5 (Tree Star, San Carlos, CA). For ICS analysis, Boolean gating was performed after single gating for each cytokine, and background from flu peptide-stimulated control samples was subtracted in Pestle (software provided by Mario Roederer, Bethesda, MD) prior to graphing. To assess functional avidity, the same ICS procedure was performed following a 5 hour, 37° incubation of cells from infected lungs or spleens with 1 µg/ml of co-stimulatory antibodies against CD28 and CD49d, 1μg/ml of monensin and serial dilutions of the indicated peptide.

### Single cell sequencing and weblogos

Single cell sequencing was performed using multiplex nested RT-PCR of the TCRβ chain from single sorted cells and data were analyzed as described previously [23]. Weblogos were generated on http://weblogo.berkeley.edu/following input of all sequences normalized to the same length by insertion of Xs into the center of shorter CDR3βs.

### Precursor frequency analysis

Single cell suspensions were isolated from the spleen and macroscopic lymph nodes (inguinal, axillary, brachial, cervical, and mesenteric) of individual mice (adults) or from the spleen only of neonatal mice (pooled spleens from 8–12 mice/sample) by manual disruption between frosted glass slides. Precursor frequencies were evaluated using an approach similar to that described for assessing naïve CD4+ T cell populations [49], [50], with other described modifications to enhance the detection of epitope-specific CD8+ T cells with less background [44], [45]. Briefly, cells were incubated in MACS buffer (Miltenyi) containing 0.1% sodium azide, Fc block (2.4G2, BD), and a labeled antibody to CD8α (53-6.7, BD). Tetramers of the same specificity labeled separately with phycoerythrin (PE) and allophycocyanin (APC) were added for one hour at room temperature. Cells were washed and incubated with 50 µL each of anti-PE and anti-APC beads for 30 minutes at 4°C before washing and passing through a magnetized MS column (Miltenyi) as directed for positive enrichment of tetramer-specific T cells. Enriched cells were resuspended in 100 µL of MACS buffer and stained with additional surface antibodies against CD3, CD4, CD44 (IM-7, e-Bioscience) and B220 (RA3-6B2, e-Bioscience), CD11b (M1/70.15, e-Bioscience), CD11c (N418, e-Bioscience), and F4/80 (BM8, Caltag-Invitrogen) antibodies were included in a dump gate that also excluded dead (ViViD+) cells. Samples were acquired in their entirety on an LSR II flow cytometer and analyzed using FlowJo software to determine the number of tetramer double-positive cells acquired from each adult mouse or pool of neonatal mice. All data are presented as number of cells obtained per mouse. Samples from naïve CB6F1 adults, naïve CB6F1 neonates, immune/memory CB6F1 adults (positive controls), and OT-I/RAG1^-/-^ mice (negative controls) were run with two sets of tetramers for each epitope: one set made in-house, and the other set obtained from Beckman Coulter.

### Adoptive transfer experiments

Cells were harvested from the spleens and macroscopic lymph nodes of naïve adult CB6F1 mice by manual disruption between frosted glass slides. Lymphocytes were harvested using Ficoll-LITE, then CD8+ T cells were purified by untouched isolation using a CD8+ T cell isolation kit (Miltenyi). Purity was assessed to be 88-95% by flow cytometry following each sort. Between 7×10^6^ and 1.5×10^7^ purified CD8+ T cells (the amount isolated from two adults for each neonate) were transferred intra-peritoneally into naïve, congenic neonates at days 4-5 of life, and infection with 2×10^6^ PFU of RSV was performed at day 7 of life. Lungs and spleens were harvested 7 days post infection and cells were isolated and stained as described previously with the addition of antibodies to Thy1.1 (HIS51, e-Bioscience) and Thy1.2 (53-2.1, e-Bioscience) to discriminate the host response from the response of adoptively transferred cells.

### Statistical analysis

Statistical analyses between two groups were conducted using a two-tailed students t test. Comparisons between multiple groups were performed in GraphPad Prism using a 1 way or 2 way ANOVA followed by Bonferroni's post-tests for multiple comparisons between all groups.

### Online supplemental material

Supplemental Figures S1 and S2 show CDR3β amino acid sequences derived from single cell sequencing of K^d^M2_82-90_ or D^b^M_187-195_ tetramer-sorted cells, respectively from infected adults and neonates. Supplemental Figures S3 and S4 show raw flow cytometry data from double tetramer sorts using tetramers made by in-house (Supplemental Figure S3) or commercially obtained from Beckman Coulter (Supplemental Figure S4). Supplemental Figure S5 shows the results of precursor frequency experiments conducted using Beckman Coulter tetramer sets. Supplemental Figure S6 shows the experimental outline, and gating strategy for naïve CD8+ T cell adoptive transfer experiments.

## Supporting Information

Figure S1 Single cell CDR3β sequences derived from CD8+ T cell populations specific for K^d^M2_82-90_at day 7 after infection of adult (left panel) or neonatal (right panel) mice.
(TIFF)

Figure S2 Single cell CDR3β sequences derived from CD8+ T cell populations specific for D^b^M_187-195_ at day 7 after infection of adult (left panel) and neonatal (right panel) mice.
(TIFF)

Figure S3 Raw flow cytometry data generated using tetramers produced in-house for precursor frequency analysis. Plots are gated on CD3+CD8+ cells.
(TIFF)

Figure S4 Raw flow cytometry data generated using commercially available tetramers (Beckman Coulter) for precursor frequency analysis. Plots are gated on CD3+CD8+ cells.
(TIFF)

Figure S5 Epitope-specific CD8+ T cell precursor frequency analysis using commercially available tetramers. (A) Precursor frequencies of epitope-specific CD8+ T cells in naïve adult and neonatal mice determined by the double tetramer enrichment method. (B) K^d^M2_82-90_/D^b^M_187-195_ precursor frequency ratios in naïve adults and neonates. (C) Epitope-specific memory precursors in adult mice infected one month previously with RSV. (D) Memory CD8+ T cell precursor ratios. Data are representative of several independent experiments with 5-12 mice/group. Error bars represent the SEM.
(TIFF)

Figure S6 Schema for adoptive transfer of naïve adult CD8+ T cells into congenic neonates prior to RSV infection and data analysis.
(TIFF)

## References

[1] Paramore LC, Ciuryla V, Ciesla G, Liu L (2004). Economic impact of respiratory syncytial virus-related illness in the US: an analysis of national databases.. Pharmacoeconomics.

[2] Hall CB, Long CE, Schnabel KC (2001). Respiratory syncytial virus infections in previously healthy working adults.. Clin Infect Dis.

[3] Glezen WP, Taber LH, Frank AL, Kasel JA (1986). Risk of primary infection and reinfection with respiratory syncytial virus.. Am J Dis Child.

[4] Shay DK, Holman RC, Newman RD, Liu LL, Stout JW (1999). Bronchiolitis-associated hospitalizations among US children, 1980-1996.. JAMA.

[5] Marchant A, Newport M (2000). Prevention of infectious diseases by neonatal and early infantile immunization: prospects for the new millennium.. Curr Opin Infect Dis.

[6] Siegrist CA (2001). Neonatal and early life vaccinology.. Vaccine.

[7] Izurieta HS, Thompson WW, Kramarz P, Shay DK, Davis RL (2000). Influenza and the rates of hospitalization for respiratory disease among infants and young children.. N Engl J Med.

[8] Barouch DH, Letvin NL (2001). CD8+ cytotoxic T lymphocyte responses to lentiviruses and herpesviruses.. Curr Opin Immunol.

[9] Guidotti LG, Chisari FV (2001). Noncytolytic control of viral infections by the innate and adaptive immune response.. Annu Rev Immunol.

[10] Mescher MF, Popescu FE, Gerner M, Hammerbeck CD, Curtsinger JM (2007). Activation-induced non-responsiveness (anergy) limits CD8 T cell responses to tumors.. Semin Cancer Biol.

[11] Yewdell JW (2006). Confronting complexity: real-world immunodominance in antiviral CD8+ T cell responses.. Immunity.

[12] Zaghouani H, Hoeman CM, Adkins B (2009). Neonatal immunity: faulty T-helpers and the shortcomings of dendritic cells.. Trends Immunol.

[13] McCarron MJ, Reen DJ (2010). Neonatal CD8+ T-cell differentiation is dependent on interleukin-12.. Hum Immunol.

[14] Garcia AM, Fadel SA, Cao S, Sarzotti M (2000). T cell immunity in neonates.. Immunol Res.

[15] Ridge JP, Fuchs EJ, Matzinger P (1996). Neonatal tolerance revisited: turning on newborn T cells with dendritic cells.. Science.

[16] Rothenberg E, Triglia D (1983). Clonal proliferation unlinked to terminal deoxynucleotidyl transferase synthesis in thymocytes of young mice.. J Immunol.

[17] Bogue M, Candeias S, Benoist C, Mathis D (1991). A special repertoire of alpha:beta T cells in neonatal mice.. EMBO J.

[18] Pannetier C, Cochet M, Darche S, Casrouge A, Zoller M (1993). The sizes of the CDR3 hypervariable regions of the murine T-cell receptor beta chains vary as a function of the recombined germ-line segments.. Proc Natl Acad Sci U S A.

[19] Martinez X, Regner M, Kovarik J, Zarei S, Hauser C (2003). CD4-independent protective cytotoxic T cells induced in early life by a non-replicative delivery system based on virus-like particles.. Virology.

[20] Zhang J, Silvestri N, Whitton JL, Hassett DE (2002). Neonates mount robust and protective adult-like CD8(+)-T-cell responses to DNA vaccines.. J Virol.

[21] Kovarik J, Bozzotti P, Love-Homan L, Pihlgren M, Davis HL (1999). CpG oligodeoxynucleotides can circumvent the Th2 polarization of neonatal responses to vaccines but may fail to fully redirect Th2 responses established by neonatal priming.. J Immunol.

[22] Rutigliano JA, Ruckwardt TJ, Martin JE, Graham BS (2007). Relative dominance of epitope-specific CD8+ T cell responses in an F1 hybrid mouse model of respiratory syncytial virus infection.. Virology.

[23] Dash P, McClaren JL, Oguin TH, 3rd, Rothwell W, Todd B (2011). Paired analysis of TCRalpha and TCRbeta chains at the single-cell level in mice.. J Clin Invest.

[24] Billam P, Bonaparte KL, Liu J, Ruckwardt TJ, Chen M (2011). T Cell receptor clonotype influences epitope hierarchy in the CD8+ T cell response to respiratory syncytial virus infection.. J Biol Chem.

[25] Price DA, Brenchley JM, Ruff LE, Betts MR, Hill BJ (2005). Avidity for antigen shapes clonal dominance in CD8+ T cell populations specific for persistent DNA viruses.. J Exp Med.

[26] PrabhuDas M, Adkins B, Gans H, King C, Levy O (2011). Challenges in infant immunity: implications for responses to infection and vaccines.. Nat Immunol.

[27] Graham BS (2011). Biological challenges and technological opportunities for respiratory syncytial virus vaccine development.. Immunol Rev.

[28] Kim HW, Canchola JG, Brandt CD, Pyles G, Chanock RM (1969). Respiratory syncytial virus disease in infants despite prior administration of antigenic inactivated vaccine.. Am J Epidemiol.

[29] Cormier SA, You D, Honnegowda S (2010). The use of a neonatal mouse model to study respiratory syncytial virus infections.. Expert Rev Anti Infect Ther.

[30] Culley FJ, Pollott J, Openshaw PJ (2002). Age at first viral infection determines the pattern of T cell-mediated disease during reinfection in adulthood.. J Exp Med.

[31] Dakhama A, Lee YM, Ohnishi H, Jing X, Balhorn A (2009). Virus-specific IgE enhances airway responsiveness on reinfection with respiratory syncytial virus in newborn mice.. J Allergy Clin Immunol.

[32] Dakhama A, Park JW, Taube C, Joetham A, Balhorn A (2005). The enhancement or prevention of airway hyperresponsiveness during reinfection with respiratory syncytial virus is critically dependent on the age at first infection and IL-13 production.. J Immunol.

[33] Tasker L, Lindsay RW, Clarke BT, Cochrane DW, Hou S (2008). Infection of mice with respiratory syncytial virus during neonatal life primes for enhanced antibody and T cell responses on secondary challenge.. Clin Exp Immunol.

[34] Tregoning JS, Yamaguchi Y, Harker J, Wang B, Openshaw PJ (2008). The role of T cells in the enhancement of respiratory syncytial virus infection severity during adult reinfection of neonatally sensitized mice.. J Virol.

[35] You D, Becnel D, Wang K, Ripple M, Daly M (2006). Exposure of neonates to respiratory syncytial virus is critical in determining subsequent airway response in adults.. Respir Res.

[36] Graham BS, Perkins MD, Wright PF, Karzon DT (1988). Primary respiratory syncytial virus infection in mice.. J Med Virol.

[37] Harker JA, Lee DC, Yamaguchi Y, Wang B, Bukreyev A (2010). Delivery of cytokines by recombinant virus in early life alters the immune response to adult lung infection.. J Virol.

[38] Stein RT, Sherrill D, Morgan WJ, Holberg CJ, Halonen M (1999). Respiratory syncytial virus in early life and risk of wheeze and allergy by age 13 years.. Lancet.

[39] Rutigliano JA, Rock MT, Johnson AK, Crowe JE, Graham BS (2005). Identification of an H-2D(b)-restricted CD8+ cytotoxic T lymphocyte epitope in the matrix protein of respiratory syncytial virus.. Virology.

[40] Ruckwardt TJ, Luongo C, Malloy AM, Liu J, Chen M (2010). Responses against a subdominant CD8+ T cell epitope protect against immunopathology caused by a dominant epitope.. J Immunol.

[41] Feeney AJ (1991). Junctional sequences of fetal T cell receptor beta chains have few N regions.. J Exp Med.

[42] Feeney AJ (1993). Junctional diversity in the absence of N regions. Neonatal T cell receptor beta chain junctional sequences are more heterogeneous than neonatal T cell receptor gamma delta or IgH junctions.. J Immunol.

[43] Rudd BD, Venturi V, Davenport MP, Nikolich-Zugich J (2011). Evolution of the antigen-specific CD8+ TCR repertoire across the life span: evidence for clonal homogenization of the old TCR repertoire.. J Immunol.

[44] Kotturi MF, Scott I, Wolfe T, Peters B, Sidney J (2008). Naive precursor frequencies and MHC binding rather than the degree of epitope diversity shape CD8+ T cell immunodominance.. J Immunol.

[45] Obar JJ, Khanna KM, Lefrancois L (2008). Endogenous naive CD8+ T cell precursor frequency regulates primary and memory responses to infection.. Immunity.

[46] Schmidt J, Neumann-Haefelin C, Altay T, Gostick E, Price DA (2011). Immunodominance of HLA-A2-Restricted Hepatitis C Virus-Specific CD8+ T Cell Responses Is Linked to Naive-Precursor Frequency.. J Virol.

[47] La Gruta NL, Rothwell WT, Cukalac T, Swan NG, Valkenburg SA (2010). Primary CTL response magnitude in mice is determined by the extent of naive T cell recruitment and subsequent clonal expansion.. J Clin Invest.

[48] Catanzaro AT, Koup RA, Roederer M, Bailer RT, Enama ME (2006). Phase 1 safety and immunogenicity evaluation of a multiclade HIV-1 candidate vaccine delivered by a replication-defective recombinant adenovirus vector.. J Infect Dis.

[49] Moon JJ, Chu HH, Hataye J, Pagan AJ, Pepper M (2009). Tracking epitope-specific T cells.. Nat Protoc.

[50] Moon JJ, Chu HH, Pepper M, McSorley SJ, Jameson SC (2007). Naive CD4(+) T cell frequency varies for different epitopes and predicts repertoire diversity and response magnitude.. Immunity.

