# Supplementary figures and images for: Neonatal CD8 T-cell Hierarchy Is Distinct from Adults and Is Influenced by Intrinsic T cell Properties in Respiratory Syncytial Virus Infected Mice
Source: PLoS Pathog. 2011 Dec 1;7(12):e1002377. doi: 10.1371/journal.ppat.1002377 (PMC3228797; doi:10.1371/journal.ppat.1002377)

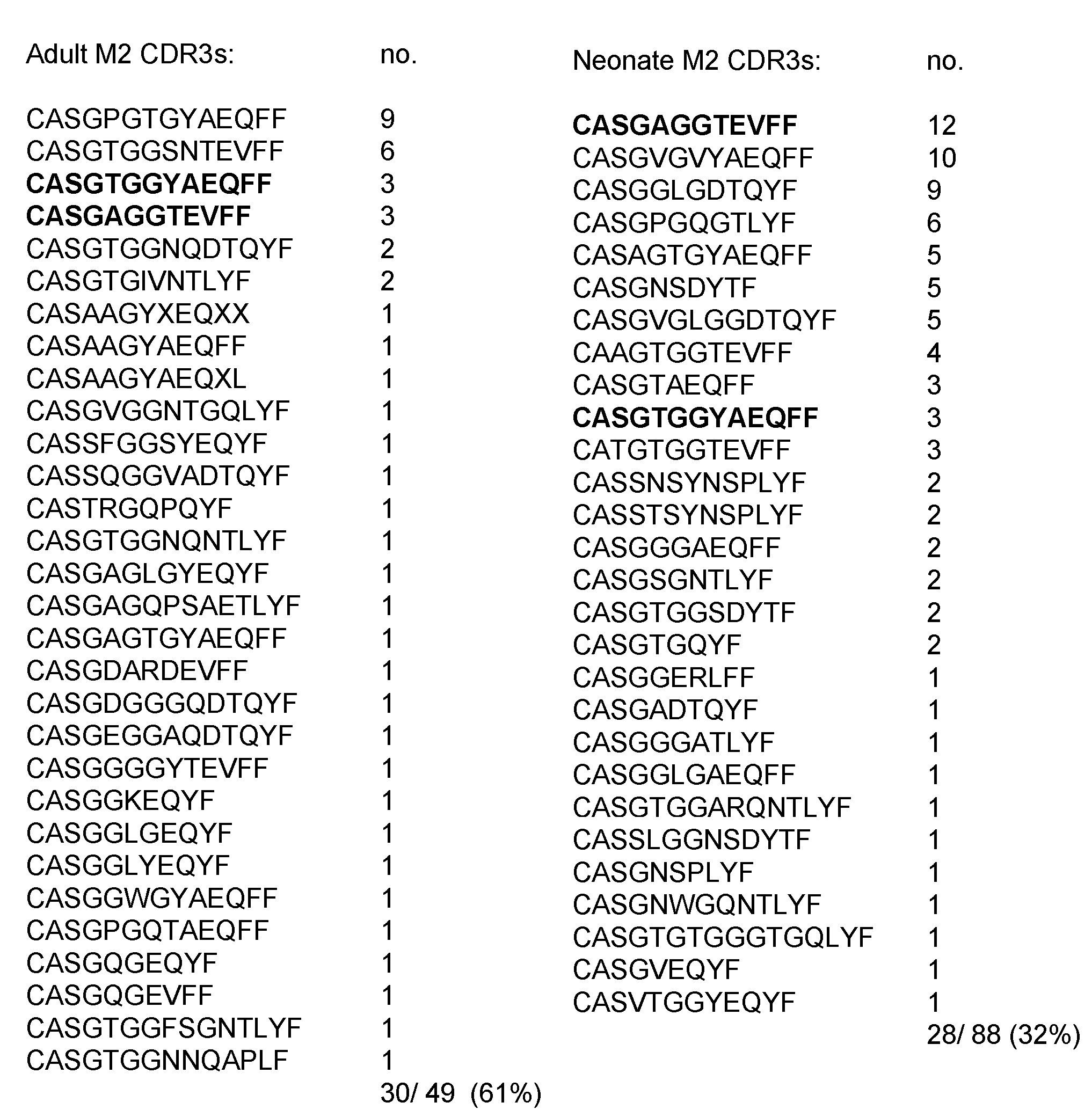

Supplement: Figure S1 — Single cell CDR3β sequences derived from CD8+ T cell populations specific for KdM282-90at day 7 after infection of adult (left panel) or neonatal (right panel) mice. (TIFF) [file ppat.1002377.s001.tiff]

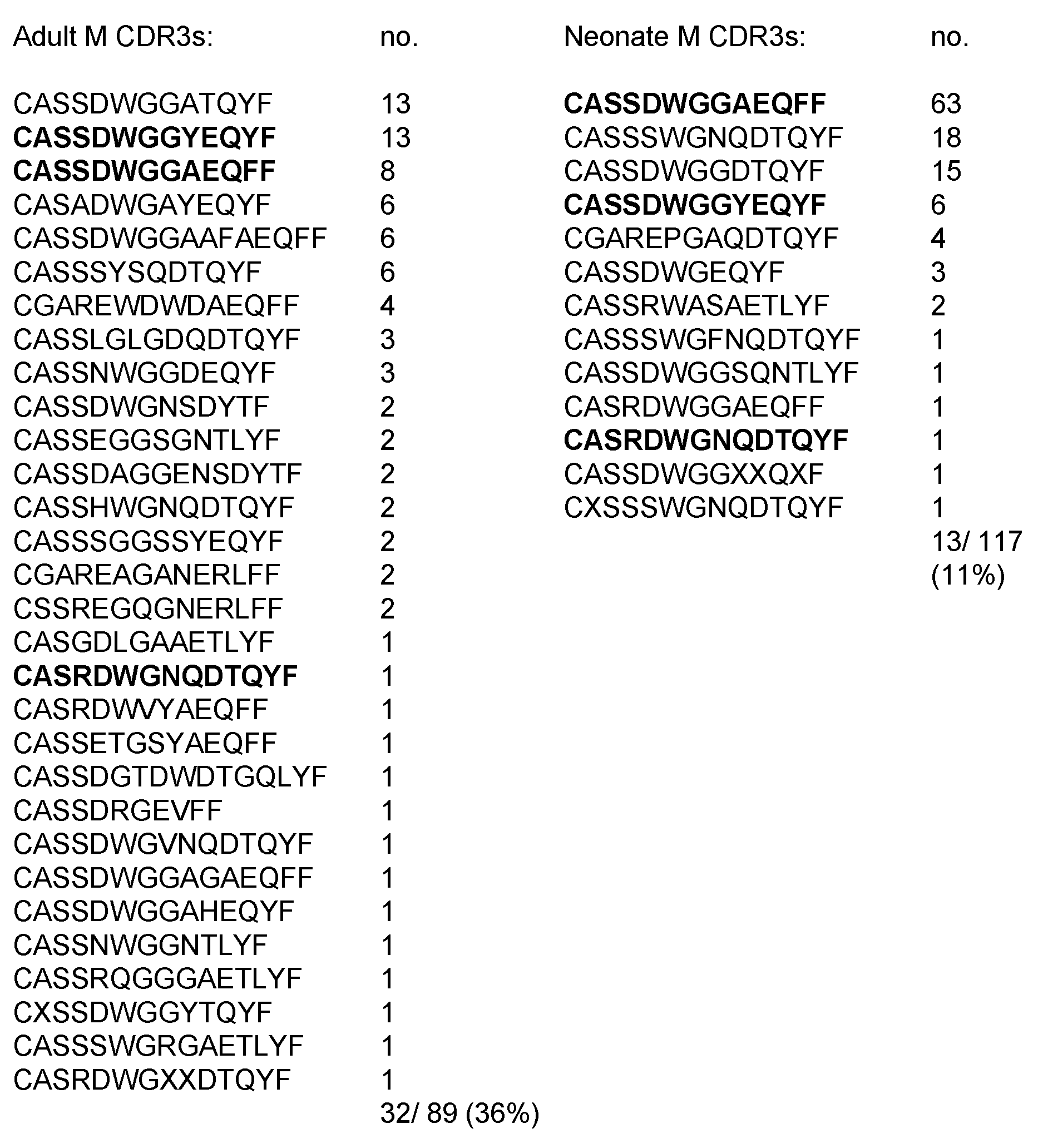

Supplement: Figure S2 — Single cell CDR3β sequences derived from CD8+ T cell populations specific for DbM187-195 at day 7 after infection of adult (left panel) and neonatal (right panel) mice. (TIFF) [file ppat.1002377.s002.tiff]

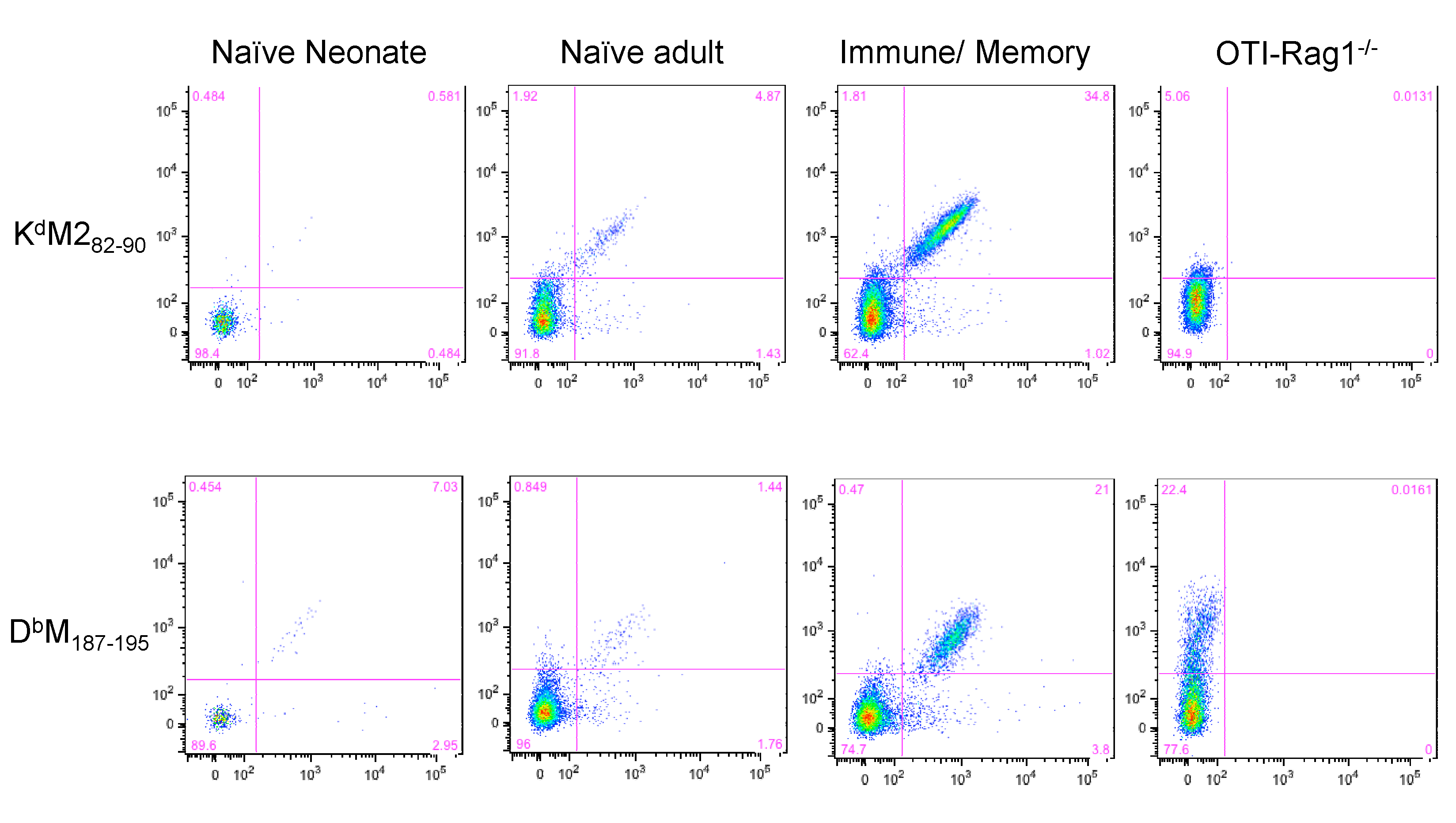

Supplement: Figure S3 — Raw flow cytometry data generated using tetramers produced in-house for precursor frequency analysis. Plots are gated on CD3+CD8+ cells. (TIFF) [file ppat.1002377.s003.tiff]

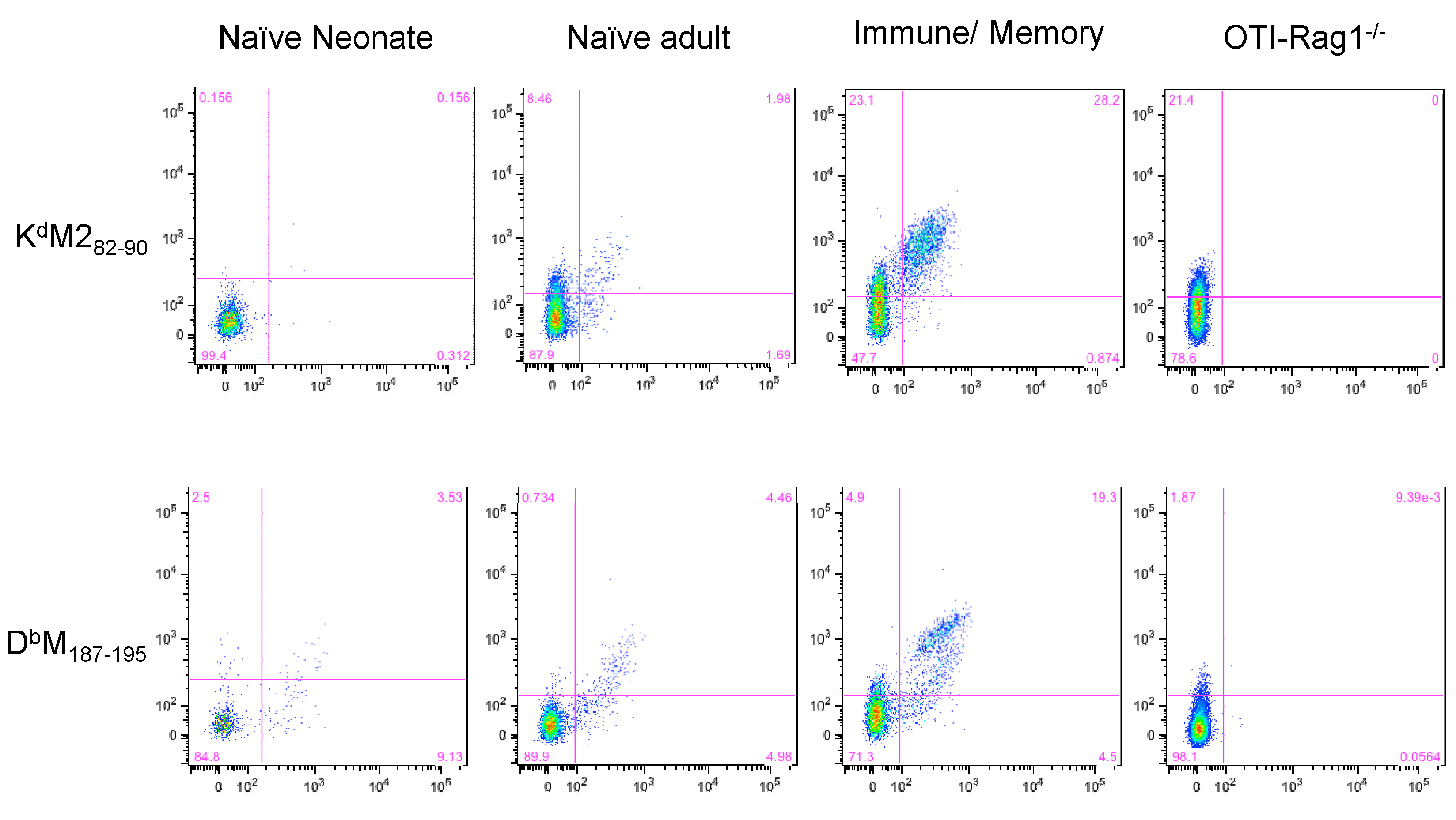

Supplement: Figure S4 — Raw flow cytometry data generated using commercially available tetramers (Beckman Coulter) for precursor frequency analysis. Plots are gated on CD3+CD8+ cells. (TIFF) [file ppat.1002377.s004.tiff]

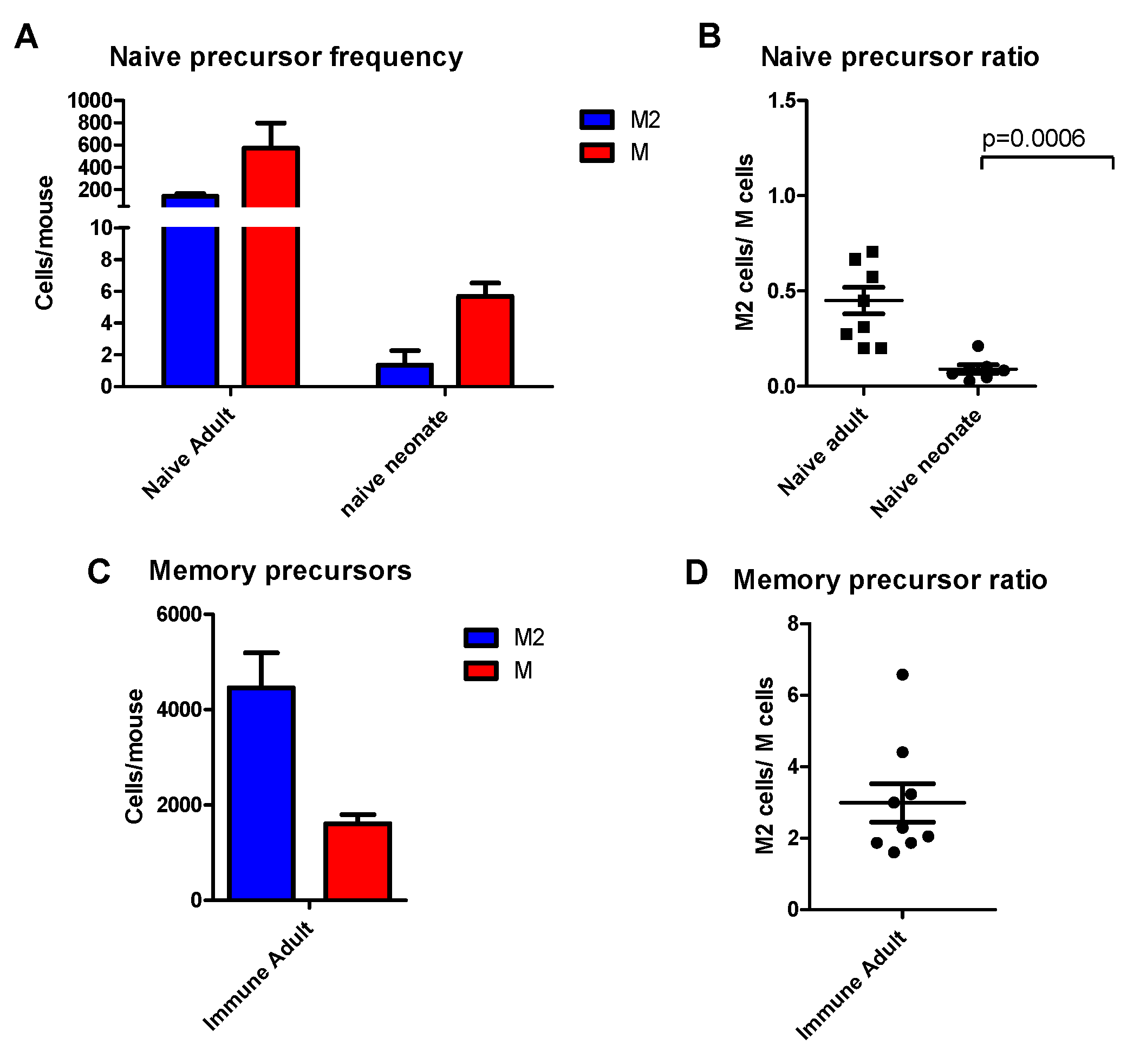

Supplement: Figure S5 — Epitope-specific CD8+ T cell precursor frequency analysis using commercially available tetramers. (A) Precursor frequencies of epitope-specific CD8+ T cells in naïve adult and neonatal mice determined by the double tetramer enrichment method. (B) KdM282-90/DbM187-195 precursor frequency ratios in naïve adults and neonates. (C) Epitope-specific memory precursors in adult mice infected one month previously with RSV. (D) Memory CD8+ T cell precursor ratios. Data are representative of several independent experiments with 5-12 mice/group. Error bars represent the SEM. (TIFF) [file ppat.1002377.s005.tiff]

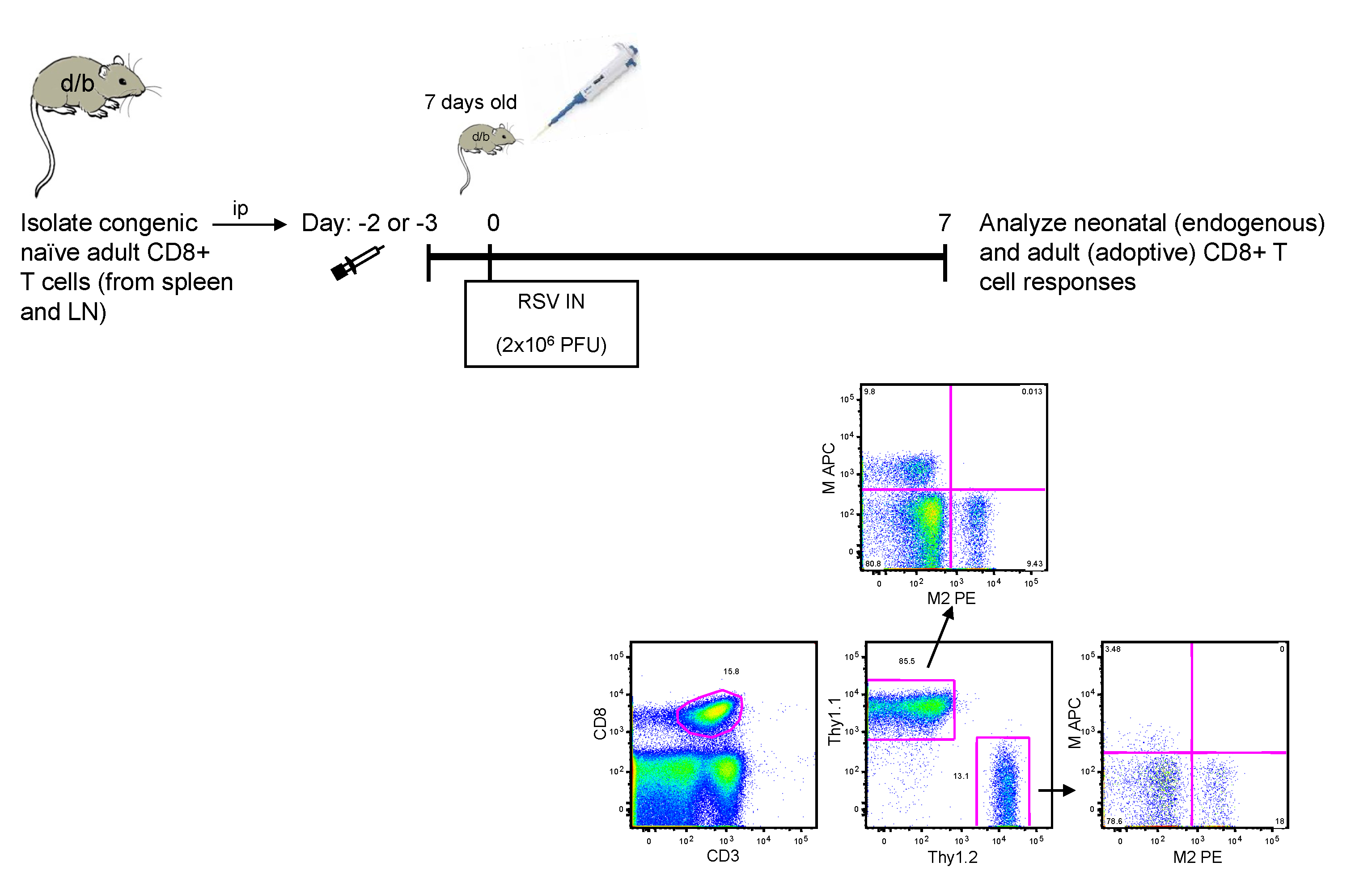

Supplement: Figure S6 — Schema for adoptive transfer of naïve adult CD8+ T cells into congenic neonates prior to RSV infection and data analysis. (TIFF) [file ppat.1002377.s006.tiff]
